# Appearance of Tumor Vessels in Patients With Choroidal Osteoma Using Swept-Source Optical Coherence Tomographic Angiography

**DOI:** 10.3389/fonc.2021.762394

**Published:** 2021-11-01

**Authors:** Nan Zhou, Xiaolin Xu, Yueming Liu, Wenbin Wei, Xianzhao Peng

**Affiliations:** ^1^ Beijing Tongren Eye Center, Beijing Key Laboratory of Intraocular Tumor Diagnosis and Treatment, Medical Artificial Intelligence Research and Verification Laboratory of the Ministry of Industry and Information Technology, Beijing Tongren Hospital, Capital Medical University, Beijing, China; ^2^ SVision Imaging, Inc., Milpitas, CA, United States

**Keywords:** choroidal osteoma, swept-source optical coherence tomographic angiography, tumor vessels, sea-fan vascular networks, terminal vascular tangles

## Abstract

**Objective:**

To report the morphologic characteristics of tumor-related vasculatures and their association with secondary choroidal neovascularization (CNV), subretinal fluid (SRF), choroidal thickness, retinal pigment epithelium (RPE) alterations, subretinal hemorrhage, and tumor decalcification in eyes with choroidal osteoma (CO), using swept-source optical coherence tomographic angiography (SS-OCTA).

**Design:**

Cross-sectional observational study.

**Participants:**

We included 26 patients recruited from Beijing Tongren Hospital with a diagnosis of CO, based on the presence of yellow-orange mass deep to the RPE under indirect ophthalmoscopy and occupying the choroid with well-defined margins and bone density on ultrasonography or computed tomography and focal hyperfluorescent spots with no homogeneous pattern on fluorescein angiography/indocyanine green angiography (FA/ICGA). Data were collected from April 1, 2020, to April 1, 2021, and analyzed from April 30 through May 30, 2021.

**Methods:**

Applying SS-OCTA systems operating at 1,050-nm wavelengths, eyes with CO were imaged.

**Main Outcome and Measures:**

Tumor-related vasculature in eyes with CO was characterized using multimodal imaging that included fundus photography, FA/ICGA, SS-OCT, and SS-OCTA, and the images were anatomically aligned. CO thickness was manually measured as the distance between the upper boundary of the tumor and the underlying sclerochoroidal interface on the SS-OCT images. Subfoveal choroidal thickness was manually measured as the distance between the Bruch membrane and the sclerochoroidal interface on the SS-OCT images.

**Results:**

Of the 26 Asian patients, 16 (62%) were women and 10 (38%) were men. The mean age was 26.8 years (median, 23; range, 8–45 years), and the mean best corrected visual acuity (BCVA) was 20/40. Thirty-three eyes underwent imaging and were diagnosed with CO. Indocyanine green angiography identified inhomogeneous hyperfluorescence due to tumor-related vasculature, and all corresponded to the structures that appeared as sea-fan vascular networks (SFVNs) combined with clusters of tangled vessels on SS-OCTA images. SFVNs were detected on SS-OCTA imaging in all eyes (100%), terminal tangled vascular structures in 32 of 33 eyes (97%), but not identified on ICGA. Of the 33 tangled vascular structures, 32 (97%) were located at the edge of or inside the tumor, and only 1 (3%) was associated with type 2 neovascularization. In addition, SS-OCT revealed SRF in 33 eyes (100%), 33 (100%) were located at the edge of CO, and only 1 was underlying macular. SRF with retinal edema was seen in 30 of 32 eyes (94%).

**Conclusions:**

In eyes with CO undergoing SS-OCTA imaging, tumor-related vasculature appears as SFVNs combined with tangled vascular structures or few type 2 neovascularization. The identification of actual tumor vasculature in patients with CO as SFVNs with inner or terminal vascular tangles rather than previously described CNV may help facilitate understanding of their pathogenesis, tumor control, and response to treatment.

## Introduction

Choroidal osteoma (CO) is a benign, rare intraocular tumor composed of mature bone (1–4), and the etiology is still unknown. Tumor blood vessels of CO were first described histopathologically by Williams in 1978 (2). At that time, there was considerable preponderance of bony structures compared with the loose fibrovascular connective tissue elements within the mass. Since the initial descriptions of CO in 1978 (2), most studies have focused on the clinical and diagnostic features of this tumor, and choroidal neovascularization was recognized as another feature of CO. It was later recognized that clinical features of CO evolved over years. Cases of tumor growth (5–9), tumor decalcification or involution (10–13), as well as methods of management of related choroidal neovascularization were documented (14–18). On the basis of multimodal imaging, indocyanine green angiography (ICGA) and structural optical coherence tomography (OCT) have shown that tumor-related vasculature of CO appeared to be focal dilatations of blood vessels of choroidal neovascular membrane (CNVM) or choroidal neovascularization (CNV), and the CNV is now described as patterns of vascular complex characterized by a large main central vessel trunk based on ICGA (19), which has been the criterion standard imaging method for diagnosis of CNV.

Although optical coherence tomography angiography (OCTA) has greatly facilitated the detection of retinal or choroidal vascular disease, such as CVNs, and has provided detailed descriptions of their structure (20), tumor-related vasculature have been poorly visualized on *en face* spectral-domain OCTA (SD-OCTA) (21, 22). Azad (23) and Ana et al. (24) described tumor-related vasculature of CO as type 1 CNVM overlying the tumor on SS-OCTA images, and Lafaut et al. (25) described tumor-related vasculature as hyperfluorescence abnormal choroidal vessels and vascular spiders present on the tumor surface on ICGA, whereas it is difficult to differentiate these choroidal vascular anomalies from tumor vasculature or subretinal neovascularization. Even in clinicopathological studies, only one definitive evidence has been provided about the anatomical structure of these tumor-related vasculatures (2). To date, the precise origin and composition of tumor vasculature have yet to be clearly described, and an accurate description is the first step toward understanding the pathophysiological mechanisms involved in CO. Thus, in our present study, we defined the tumor-related vasculature, including tumor vasculature and secondary choroidal neovascularization of CO. Furthermore, observations in this study using SS-OCTA (in-tissue depth 2.7 mm) have suggested that previously, the use of the term “secondary CNV” does not accurately describe the clinical appearance of these tumor-related vasculature in CO.

With a fast-tuning laser source and balance detection, Swept Source OCT (SS-OCT) can achieve higher spectral resolution than SD-OCT and therefore leads to higher imaging depth. Besides, SS-OCT exhibits less sensitivity roll-off with imaging depth because of its immunity from fringes washout effect of SD-OCT platforms. The wavelength of the swept source is usually longer (1,050 nm for retina imaging) than the light source of SD-OCT (840 nm) and results in deeper penetration through the retinal pigment epithelium (RPE) (26–28). As a result, the structural and angiographic images of SS-OCT appear superior to SD-OCT images. Therefore, the SS-OCT platform allows for better visualization of neovascularization (27) and the tumor-related vasculature. Using SS-OCTA, we investigated the morphologic characteristics of tumor-related vasculature, sea-fan vascular networks (SFVNs), and their spatial associations in a Chinese population with CO.

## Methods

This cross-sectional study included patients from the Beijing Tongren Eye Center, Beijing Tongren Hospital, Beijing, China. The patients were evaluated from April 1, 2020, to April 1, 2021. The study and data collection were compliant with the principles of the Declaration of Helsinki, and written informed consent was obtained from all participants. The study was approved by the Medical Ethics Committee of the Beijing Tongren Hospital. Inclusion criteria for the study were examined by at least one senior ophthalmologist (WW).

CO was defined as a yellow-orange mass deep to the RPE and occupying the choroid with well-defined margins and bone density on ultrasonography or computed tomography (29, 30). Exclusion criteria were severe media opacity that prevented adequate ICGA or OCTA examinations and cases of sclerochoroidal calcification (31, 32), a condition often confused with CO. All patients were in good health systemically. Anti-vascular endothelial growth factor (Anti-VEGF) therapy was offered to treat retinal edema, SRF, and the supposed choroidal neovascularization associated with the tumor.

The demographic data included patient age at diagnosis (years), sex, and race/ethnicity (Chinese, Asian). All patients underwent a complete ophthalmologic examination, including review of medical records, best-corrected visual acuity (BCVA), fundus photography (Imagenet 6, Topcon, Japan; CLARUS 500; Carol Zeiss), simultaneous FA and ICGA (Spectralis; Heidelberg Engineering, Inc), SS-OCT, and SS-OCTA (SS-OCT, VG200D, SVision Imaging, Ltd., China). The detailed system parameters of this SS-OCTA system including: central wavelength: 1050nm; spectral width: 110nm (range: 990-1100nm); A-line speed: 200K A Scans per second; transverse resolution: 15μm (optical); longitudinal resolution: 5μm (optical); and phase jitter: 20-70pm (before correction).

We defined the term sea-fan vascular networks (SFVNs) with inner or terminal tangled vascular structures detected on SS-OCTA to describe the intratumoral vasculature of CO. The SS-OCTA was performed at the same visit as the ICGA in all patients before any treatment or observation decisions were made. The choroidal thickness was manually measured as the subfoveal distance between the Bruch membrane and the sclerochoroidal interface using structural sectional OCT images. SS-OCTA was performed using 3 × 3 mm and 6 × 6 mm macular raster scans centered on the lesion in all cases. For *en face* imaging, a custom segmentation strategy was used first to visualize the tumor-related vasculature. The inner boundary followed the RPE, and the outer boundary followed the Bruch membrane, also known as the RPE-fit boundary layer on the instrument. The alternative custom segmentation strategy was the upper boundary followed the surface of the tumor, and the lower boundary followed the interface between tumor and sclera. The segmentation boundaries were then manually adjusted to optimally visualize the tumor-related vasculature, and terminal or inner tangled vessels. The data collected from each patient included their history of eye diseases, treatments, choroidal thickness measurements, and interpretations of their fundus photographic, fluorescein angiographic, ICGA, and SS-OCT images. The 3 × 3 mm, 6 × 6 mm SS-OCTA images were overlaid on the 12 × 12 mm images and magnified ICGA images to determine the position of tumor vasculature or CNV.

### Statistical Analysis

Data were analyzed from April 30 through May 30, 2021. Two ophthalmologists (NZ and XX) and one intraocular oncologist (WW) evaluated the lesions. The ophthalmologists marked and adjusted manually the segmentation boundaries of the tumor lesions (33) and tumor-related vasculature independently, first on SS-OCTA images and then adjusted to ICGA images, and the intraocular oncologist adjudicated any discrepancies. In this study, the SS-OCTA images were edited and evaluated on the point-by-point manually aligned B-scan images. All the retinal vascular projections in SS-OCTA images were removed to eliminate the artifact of retinal blood flow within the slab that would complicate the interpretation of choroidal new vessels and structures within the tumor-related vasculature. This process used the automated projection-artifact removal software that was integrated with the SVision Imaging instrument (34).

The demographics and tumor characteristics of patients with CO were summarized as follows. Data collected on continuous scale, including age (years), largest tumor basal diameter (mm), tumor thickness (mm), and choroidal thickness (μm), pre- or post-treatment were expressed as mean, median, minimum, and maximum, and they were evaluated with Student’s *t*-test, Wilcoxon matched-pairs signed-ranks test, and the chi-squared test, which were used when appropriate for determining differences in cross-sectional characteristics. A two-sided *p*-value <0.05 was considered for statistical significance. All analyses were performed in Stata (15.0).

## Results

The 26 Asian patients included 16 (62%) women and 10 (38%) men. The mean age was 26.8 years (median, 23; range, 8–45), and mean BCVA was 20/40. Thirty-three eyes underwent imaging and were diagnosed with CO. There were 19 patients (73%) who had unilateral involvement and 7 patients (23%) who had bilateral tumors. Macular involvement with tumor was seen in 5 eyes (15%) and juxtapapillary in 28 eyes (85%). The mean tumor basal diameter was 15.6 mm (median, 10.5; range, 2.4–20.1), and the mean tumor thickness was 1083.2 μm (median, 1125.6; range, 290.1–2,227.9). The mean largest choroidal thickness (LCT) was 556.5 μm (median, 519.5; range, 231.3–694.1) in affected eyes and 488.9 μm (median, 511.7; range, 207.4–646.5) in fellow eyes. Demographic and clinical characteristics of patients are summarized in **Table 1**. A total of 8 eyes were treatment-naive, 22 eyes had received multiple injections of VEGF inhibitors, and 3 eyes underwent multiple anti-VEGF treatments and photodynamic therapy. The LCT pre- and post-anti-VEGF treatment was 476.7 μm (median, 498.0; range, 236.1–694.1) and 457.3 μm (median, 477.7; range, 230.1–692.3), respectively. There was no significance difference between them (*p* < 0.05).

**Table 1:** Demographic and clinical characteristics of patients with choroidal osteoma (CO).

Patient/gender	Diagnostic age	Eye	Tumor location	Treatment	Tumor thickness (μm)	LCT—before treatment (μm)	LCT—after treatment (μm)	The fellow eye
1/M	37	OU	Peripapillary	3 Anti-VEGF injections (OU)	494.04/545.2	261.0/236.1	258.2/237.1	
2/FM	14	R	Peripapillary	2 Anti-VEGF injections	290.1	421.4	382.2	207.4
3/FM	33	R	Macular	3 Anti-VEGF injections (OU)	1143.8	689	692.3	646.5
4/M	28	L	Peripapillary	7 Anti-VEGF injections and 3 PDT	376.2	694.1	638.1	655.4
5/M	25	OU	Peripapillary	None	515.8/558.2	525.7/512.4		
6/M	25	L	Peripapillary	3 Anti-VEGF injections	1128	525.2	502.3	533.1
7/FM	30	R	Peripapillary-macular	12 Anti-VEGF injections and 3 PDT	1391.3	697.6	671.5	494.1
8/FM	25	R	Peripapillary	6 Anti-VEGF injections and 3 PDT	1299.8	556.8/511.9	511.7/499.7	522.6
9/FM	18	R	Peripapillary	12 Anti-VEGF injections	1108.8	633.9	602.6	611.4
10/FM	27	OU	Peripapillary	3 Anti-VEGF injections (OU)	1056.7/1125.6	545.7/578.6	521.6/533.6	
11/FM	36	L	Macular	2 Anti-VEGF injections	1446.6	296.7	281.2	388.1
12/FM	45	OU	Peripapillary	4 Anti-VEGF injections	1332.6/118.4	443.6/411.6	409.4/398.6	
13/M	30	OU	Peripapillary	2 Anti-VEGF injections	1722.6/1168.5	512.6/508.9	501.3/478.9	
14/FM	15	OU	Peripapillary	12 Anti-VEGF injections (OU)	1010.6/835.0	487.0/545.4	476.5/533.6	
15/FM	25	OU	Peripapillary	3 Anti-VEGF injections and 2 PDT	1507.8/1275.7	368.8/401.3	338.9/382.7	
16/FM	36	R	Macular	2 Anti-VEGF injections	688.8	376.8	354.6	290.1
17/M	37	L	Peripapillary	3 Anti-VEGF injections	789.6	412.6	402.1	306.7
18/M	38	R	Peripapillary	4 Anti-VEGF injections	988.6	694.1	608.2	621.5
19/M	25	L	Peripapillary	5 Anti-VEGF injections	1149.7	511.4	483.2	511.7
20/M	16	R	Peripapillary	3 Anti-VEGF injections	1713.4	427.5	406.5	513.2
21/FM	11	L	Peripapillary	None	861.6	557.2		521.6
22/FM	8	R	Peripapillary	None	1354.3	299.1		432.2
23/FM	28	R	Peripapillary	2 Anti-VEGF injections	2227.9	396	327.7	468.5
24/FM	21	R	Peripapillary	3 Anti-VEGF injections and 2 PDT	2136.6	388.1	353.6	432.5
25/FM	20	L	Macular	3 PDT and 2 KLP	984.8	231.3	230.1	449.1
26/M	25	L	Peripapillary	2 Anti-VEGF injections	1397.6	571.6	516.6	588.4

LCT, largest choroidal thickness; PDT, photodynamic therapy; KLP, krypton lasers photocoagulation.

Sea-fan vascular networks were detected on SS-OCTA in all 33 eyes (100%), and ICGA detected CNV in 1 of 33 eyes (3%). All SFVNs located within the CO, covering the calcified and decalcified areas of the tumor. In 33 eyes, the SFVNs that could not be clearly identified on ICGA were detected on SS-OCTA. By adjusting the segmentation boundaries to interpret the SS-OCTA images, we were able to identify all the tumor-related vasculature.

**Figure 1:**
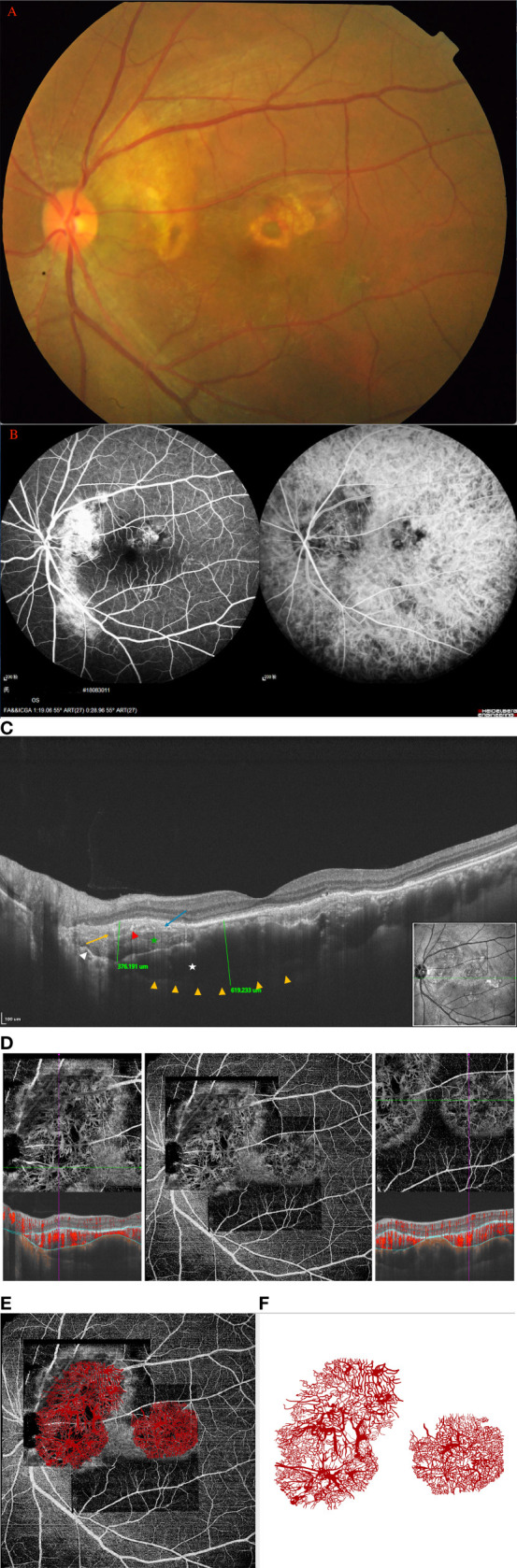
(Patient 4) **(A)** Fundus examination revealed two orange-red lesions in the posterior fundus, one located at peripapillary with partial decalcification, another small lesion in the macula area. **(B)** FA/ICGA revealed that the hypofluorescent area observed in the early phase corresponds to the extent of osteoma but the borders may be difficult to demarcate, and diffuse mild hyperfluorescence in late phase. **(C)** On the SS-OCT, B-scan revealed a 376-μm-thick domed tumor and 619-μm-thick choroidal thickness, respectively. 16 mm B-Scan: white star: trabecular bone; green star: denser and striated cortical bone; long orange arrow: Haversian or Volkmann vascular channels; red arrowheads: hyperreflective dots; blue arrow: alteration of external retinal layers and RPE above the tumor; white arrow: the external choroid seems pushed toward the outside and compressed. **(D)** The SS-OCTA boundary segmentation showed that the SFVNs and terminal vessels appeared intertwined with tangled vascular structures that corresponded to a tumor vessel on B-scans. **(E)** The En-face Retinal Depth Encoded of the 6 × 6 mm SS-OCTA cube scan demonstrated two areas of quiescent tumor vasculatures with no exudative sign found on the B-scan. The vascular network is sea-fan-shaped with small vascular tangles, the terminal vessels of the vascular network are thinner, and the terminal vessels are thin, little showing angiomatous dilation. The tumor-related vasculature appeared to be composed of sea-fan vascular networks and numerous tangle vessels when the lesions were magnified, as outlined in our schematic drawing **(F)**, which is consistent with the tumor-related vasculature on ICGA **(B)**.

The internal blood flow of the tumor-related vasculature found on ICGA appeared as vascular complex, and sea-fan like vascular networks with inner or terminal tangled vascular structures on SS-OCTA. SS-OCT revealed SRF in 33 eyes (100%); 32 (97%) were located at the edge of CO, and only 1 eye was underlying macular. SRF with retinal edema was seen in 30 of 32 eyes (94%). In addition, a few terminal vessels of the vascular network similar to tangled vascular structures were detected on SS-OCTA that were connected to flat irregular SRFs on OCT B-scans (**Figure 4** in the Supplement) but not clearly detected on ICGA (**Figure 1**). The tangled vascular structures were located within SFVNs, and only one (3%) was connected with type 2 neovascularization. The *en face* images of these tumor-related vasculatures appeared as sea-fan, radial, branched, cluster, or other irregular shapes, not showing telangiectasis. Tangled vascular structures were within SFVNs in all 33 eyes but also at the border of SFVNs in 2 eyes.

### Patient 4

A 28-year-old man diagnosed with CO on the left eye received 12 anti-VEGF treatments (ranibizumab [Lucentis; Novartis], 0.5 mg) for 5 years. BCVA was 16/20 OD and 20/25 OS, respectively. Fundus examination revealed two orange-red lesions in the posterior fundus, one located at peripapillary with partial decalcification and another small lesion in the macula area (**Figure 1A**). ICGA revealed that a hypofluorescent area observed in the early phase corresponds to the extent of the osteoma but the borders may be difficult to demarcate, and there was diffuse mild hyperfluorescence in the late phase (**Figure 1B**). On the SS-OCT, B-scan revealed a 376-μm-thick domed tumor (**Figure 1C**). The largest choroidal thickness was 619.2 μm in affected eyes, and 633.9 μm in the fellow eye. The outer retina had thinned, with alteration of the RPE. The heterogenous aspect within the tumor-associated hyper- and hyporeflectivity is similar to trabecular bone as described by Williams et al. on histological sections (2). The densest adjacent areas resembled cortical bone, within which a striated lamellar aspect was found, possibly cement lines. Several hyperreflective dots were found within both types of bones. Moreover, hyporeflective tubular areas could correspond to bone vascular channels (Volkmann or Haversian channels), as described by Shields et al. (5), even though in SS-OCTA, no flow was detected in these canals (**Figure 1C**). The En-face Retinal Depth Encoded of the 6 × 6 mm SS-OCTA cube scan (**Figure 1D**) demonstrated two areas of quiescent tumor vasculatures with no exudative sign found on the B-scan. The analysis of SS-OCTA flow showed the SFVNs and tangled vascular vasculature in which dense flow signals were found (**Figure 1E**). The SS-OCTA boundary segmentation showed that the SFVNs and terminal vessels appeared intertwined with tangled vascular structures that corresponded to a tumor vessel on B-scans (**Figure 1D**). Furthermore, the tumor-related vasculature appeared to be composed of sea-fan vascular networks and numerous tangle vessels when the lesions were magnified (**Figure 1E**), as outlined in our schematic drawing (**Figure 1F**), which is consistent with the tumor-related vasculature on ICGA. After anti-VEGF therapy, the SS-OCTA *en face* image showed no reduction in the size of the SFVN and vascular tangles (**eFigures 1A**–**F** in the Supplement). The terminal vascular tangles were observed to recur 2 months after anti-VEGF therapy, and the tangled vascular structure seemed more distinct in some tumor-related vasculature (e.g., the tumor-related vasculature numbered A and B in **eFigure 1G** in the Supplement). Four months after anti-VEGF therapy, denser vascular tangles were observed at the end of SFVNs. They were associated with some newly formed lesions and the growth of tumor (e.g., the tumor-growth numbered A and B **eFigure 1H** in the Supplement). This case demonstrated that tumor-related vasculature resembled a lesion consisting of SFVNs and vascular tangles rather than secondary choroidal neovascularization that arose from a choroid.

### Patient 2

**Figure 2:**
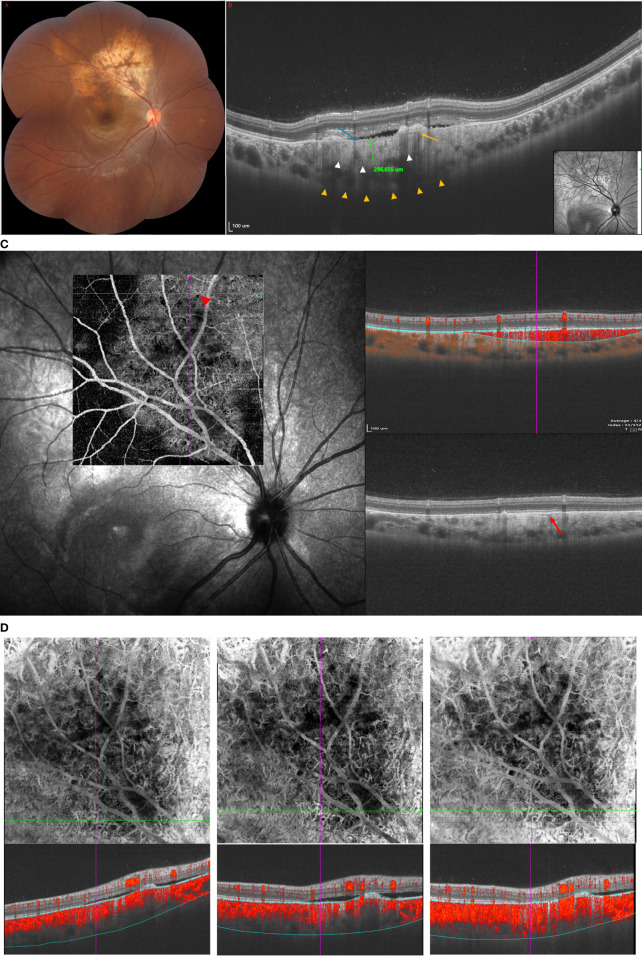
(Patient 2) **(A)** Fundus examination revealed a yellowish-orange lesion located at peripapillary associated with a local SRF involved macular. **(B)** On the SS-OCT, B-scan revealed a 296-μm-thick flat osteoma in the early stage. 16 mm B-Scan: long orange arrows: Haversian or Volkmann vascular channels; blue arrow: alteration of external retinal layers and RPE above the tumor; white arrow: the external choroid seems pushed toward the outside and compressed. The trabecular bone, dense bone, and striated bone were not demonstrated in the early stage of the tumor. **(C)** The SFVNs and tangled vascular structures of tumor-related vasculature were clearly seen on SS-OCTA before and after anti-VEGF treatment. **(D)** Two months after the second ranibizumab (0.5 mg) injection, SS-OCTA showed regression of SRF, but no reduction of the terminal vessels in the tumor-related vasculature that appeared as a dilated vessel connected to the SFVN.

A 14-year-old girl was diagnosed with CO on the left eye for 1 year and had received three ranibizumab (0.5 mg) injections 1 year previously. She experienced gradual deterioration in the vision of her left eye. BCVA was 20/25 OS. Fundus examination revealed a yellowish-orange lesion located at peripapillary associated with a local SRF involved macular (**Figure 2A**). Early-phase FA showed evidence of focal hyperfluorescence, and late-phase FA showed leakage with a pooling configuration that suggested the diagnosis of active retinal serous detachment (**eFigure 2A** in the Supplement). ICGA revealed focal hyperfluorescence with SFVNs in the early frames (**eFigure 2A** in the Supplement). On the SS-OCT, B-scan revealed a 296-μm-thick flat tumor (**Figure 2B**). The largest choroidal thickness was 383.2 μm in the affected eyes, and 207.4 μm in the fellow eye. The SFVNs and tangled vascular structures of tumor-related vasculature were clearly seen on SS-OCTA before and after anti-VEGF treatment (**Figure 2C**). Two months after the second ranibizumab (0.5-mg) injection, SS-OCTA showed regression of SRF, but no reduction of the terminal vessels in the tumor-related vasculature that appeared as a dilated vessel connected to the SFVN (**Figure 2D**). This case showed that the tumor-related vasculature consisted of sea-fan vascular networks combined with tangled vessels, and anti-VEGF treatment was associated with regression of SRF secondary from tumor vascular structures (**eFigure 2B** in the Supplement). In addition, we found that patients with CO received anti-VEGF treatment, and with the absorption of subretinal fluid, the choroidal thickness (“pachychoroid”) of the affected eye showed reduction or became thinner (**eFigure 2B** in the Supplement).

### Patient 14

**Figure 3:**
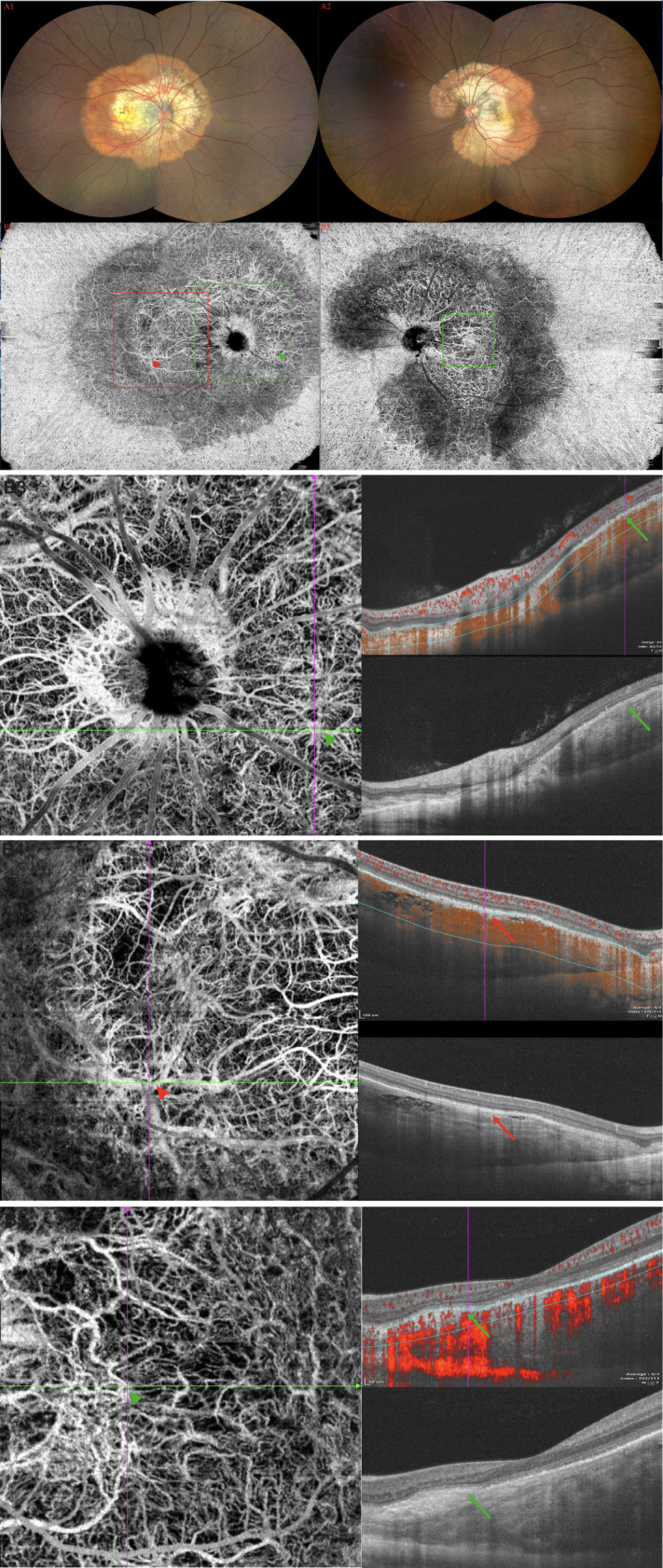
(Patient 14) **(A1, 2)** Fundus examination revealed large diffused yellowish-orange lesions in the posterior fundus involved the macula, with partial decalcification. **(B1, 2)** SS-OCTA revealed SFVNs with tangled vascular structures that corresponded to the tumor-related vasculature seen on FA/ICGA (arrows). The En-face Montage images of the 23.5 × 17.5 mm SS-OCTA exhibited two large areas of quiescent tumor vasculature with no exudative sign found on the B-scan; the tumor-related vasculature appeared to be composed of sea-fan vascular networks and numerous tangle vessels when the lesions were magnified as 6 × 6 mm and 3 × 3 mm **(B3–5)**, respectively. The caliber of tumor vessels is commonly one-eighth to one-quarter that of a major retinal vein at the disc margin, and occasionally they are as large as such veins. The tumor blood vessels frequently form networks that often resemble part or all of a carriage wheel. The vessels radiate like spokes from the center of the complex to a circumferential vessel bounding its periphery. SFVNs vessel networks may also be irregular in shape, without a distinct radial pattern.

A 15-year-old girl experienced chronic visual loss from bilateral eyes. BCVA was 16/20 OD and 20/25 (treatment naive), respectively. Fundus examination revealed large diffused yellowish-orange lesions in the posterior fundus involved the macula, with partial decalcification (**Figures 3A1, 2**). FA showed evidence of early focal hyperfluorescence with late leakage, ICGA revealed patchy hyperfluorescence in the early frames (**eFigures 3A1, 2** in the Supplement). On the SS-OCT, B-scan revealed a 1,507.8-μm-thick diffused tumor on OS and 1275.7-μm-thick diffused tumor on OD (**eFigures 3B1, 2** in the Supplement). SS-OCTA revealed SFVNs with tangled vascular structures that corresponded to the tumor-related vasculature seen on ICGA. The En-face Montage images of the 23.5 × 17.5 mm SS-OCTA (**Figures 3B1, 2**) exhibited two large areas of quiescent tumor vasculature with no exudative sign found on the B-scan, and the tumor-related vasculature appeared to be composed of sea-fan vascular networks and numerous tangle vessels when the lesions were magnified as 6 × 6 mm and 3 × 3 mm (**Figures 3B3–5**), respectively. The Montage image of superficial retinal vascular layer showed that the retinal vasculature were normal (**eFigures 3C1, 2** in the Supplement). The SFVNs and tiny tangled vessels were not reduced after 13 anti-VEGF therapies; however, the size of tumors remained stable and the SRF did not recur.

### Patient 3

**Figure 4:**
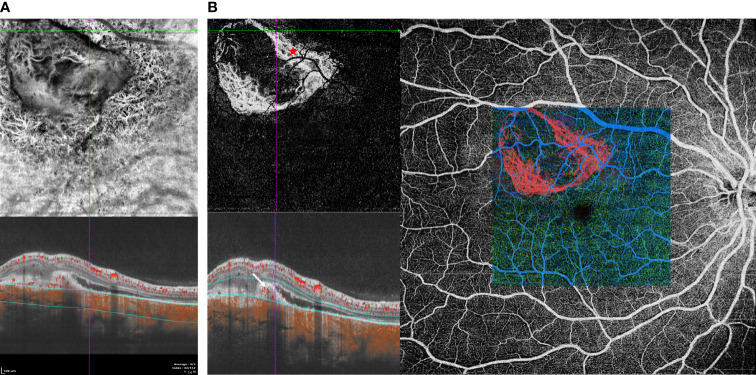
(Patient 3) **(A)** SS-OCTA showed SFVNs with tangled vascular structures that corresponded to the tumor-related vasculature seen on FA/ICGA (**eFigure 4B**). **(B)** Type 2 neovascularization and tumor-related vasculature were seen when the boundary layers on the cross-sectional SS-OCTA B-scan transitioned from the top of the lesion to choroid.

A 33-year-old woman experienced acute visual loss from her right eye. BCVA was 40/100 OD (treatment naive). Fundus examination revealed diffused reddish-orange lesion in the superior macula (**eFigure 4A** in the Supplement). FA showed evidence of early focal hyperfluorescence with late leakage consistent with type 2 neovascularization (**eFigure 4B** in the Supplement). ICGA revealed focal hyperfluorescence in the early frame (**eFigure 4B** in the Supplement). SS-OCTA showed SFVNs with tangled vascular structures that corresponded to the tumor-related vasculature seen on ICGA (**Figure 4A**). Type 2 neovascularization and tumor-related vasculature were seen when the boundary layers on the cross-sectional SS-OCTA B-scan transitioned from the top of the lesion to choroid (**Figure 4B**). This case demonstrated that SFVN and type 2 neovascularization coexisted in the same eye, and tumor vasculature may have been derived from one or both of these neovascular lesions, as outlined in our schematic drawing (**eFigures 4C1, 2** in the Supplement).

## Discussion

In the present study, SS-OCTA identified SFVNs and tumor vasculature in CO better than ICGA, and SS-OCTA revealed the appearance of tumor vasculature as SFVNs with tangled vascular structures that have always been identified as type 1 or type 2 CNV in patients with CO. The observation that tumor vasculature appears to be SFVNs and tangled vascular structures differs from the proposal that tumor-related vasculature of CO is type 1 or type 2 CNV of neovascular tissue and similar to aneurysms in the AMD or PCV (5, 19). Several independent clinicopathological investigations (1, 2) have shown that between the bony trabecula, there were large, blood-filled cavernous vascular spaces as well as small capillary-type blood vessels, and these vascular structures were lined by a single layer of flat endothelial cells. The SFVNs of CO were tumor vascular in nature and it is speculated (proposed) that the tumor vasculature may associate with the inner choroidal circulation. However, the SFVNs have not been definitely detected in the histopathologic examinations.

Our SS-OCTA observations suggest that the tumor-related vasculature on ICGA consists mostly of the SFVN vascular complex. Most of the tumor-related vasculature were found to consist of SFVNs combined with tangled vessels clusters, such as a tiny curled glomerular-type lesion, rather than a single CNV. The findings are consistent with the tumor vascular structure reported in the study of Williams et al. (2), in which they described “large, blood-filled cavernous vascular spaces as well as small capillary-type blood vessels.” on histopathology. Because of the benign nature and extreme lack of tissues, and relatively lower image quality of histopathological findings, they were unable to describe more detailed information about the tumor-related vasculature. Lafaut et al. (25) reported that “the findings on fluorescein and ICG angiography in CO are complex and heterogeneous” and included vascular spiders and abnormal choroidal vessels, with no homogeneous patterns, suggesting that the tumor-related vasculature has different variants in nature. None of these studies described tumor vasculature as a sea-fan-like vascular network inside CO. We also observed little variants of tumor vasculature on SS-OCTA; the flow signal from the tumor vasculature appeared consistent with that of SFVNs and abnormally tangled vessels that could be focally dilated and form small loose or dense globular structures, presenting different shapes and sizes.

Previous studies (35–38) indicated that the treatment with anti-VEGFs may influence the CNV (and maybe tumor) network. In our series, 22 of 33 eyes had received multiple injections of VEGF inhibitors, and the tumor-related vasculature also appears to respond to anti-VEGF therapy with the regression of SRF (**Figure 3**), while remaining stable in size and complexity. These findings are also consistent with the observations by other intraocular tumors that partly minor tumor-related vasculature disappeared after anti-VEGF treatment (35, 36). This finding is consistent with the observations that in neovascular age-related macular degeneration, the complexity of the neovascularization diminishes after anti-VEGF therapy, whereas the larger, more mature vessels are unchanged (37, 38). Moreover, the main trunk blood vessels of SFVNs would not be expected to respond to anti-VEGF treatment, whereas a terminal tangle of new vessels adjacent to edge of tumor is likely to respond, which may be attributed to the regression of SRF. Perhaps, SS-OCTA of tumor-related vasculature will provide important clues about classifying tumor-related vasculature as active or quiescent in CO, and the vascular complexity of these tumor-related vasculature may provide factors of tumor growth and prognostic indications of whether they will respond to anti-VEGF therapy or recur after treatment.

The belief that tumor-related vasculature of CO represented secondary CNV lesions is based on the phenomenon of dye washout on ICGA and neovascular-like lesions on avascular layer on SD-OCTA and CNVM on SS-OCTA (23–25). However, these features could also be found within a tangled vascular structure, especially on PCV. Moreover, the SFVNs and tangled vessels were seen in all tumor-related vasculature, whereas the ICGA phenomenon described above was not observed in all tumor-related vasculature, indicating that tumor-related vasculature is a uniform entity and has little variety of configurations. We propose that the lack of variability of SFVNs is based on the benign tumor in nature and the relative maturity of the tumor vessels in CO, not in the configuration or the complexity of the tumor vascular tangles. In addition, SS-OCTA showed that the SFVNs and partial terminal tumor-related vasculature may communicate or is associated to the choroidal vasculature; thus, the thinner-walled dilated vessels at the edge of the tumor vasculature may display blood flow signals. We also found that the tangled vessels appeared to be derived from the existing SFVNs and were arranged in a fine loop or whorl pattern or in a cluster networks of vessels or bunch-of-grapes pattern. The sea-fan pattern would be consistent with the views of Lafaut et al. (25) that the tangled vessels would be visible as vascular spiders of hyperfluorescence on ICGA as the dye intensity fades from the central lumens during the washout period. Finally, we also support the notion of turbulent flow within these SRVNs and the tangled vessels because of the presence of dilated vessels of varying caliber. The presence of vascular dilation and changes in vascular caliber and direction within the tangled vascular networks may contribute to turbulent flow.

Previous SD-OCT and post-enucleation studies have located tumor-related vasculature within the tumor, not above the Bruch membrane and beneath the RPE (2, 39). We identified all the tumor-related vasculature consisting of SFVNs and tangled vessels as located in the inner tumor not outside the area between the Bruch membrane and RPE layer (type 1 or type 2 neovascularizations) in 33 eyes (100%) and type 2 neovascularization at the margins of SFVN in 1 eye (3%). We also observed that tumor vasculature coexisted with type 2 neovascularization in the same eye, which further suggests that SFVNs are tumor vascular structures, rather than aneurysmal variations of type 1 or type 2 CNV secondary to CO.

To date, the criterion standard for the diagnosis of tumor-related vasculature is CNV associated to CO by ICGA or SD-OCT, which we now question, given the findings of several studies in which the detectability of tumor vessels was confirmed with histopathology, and the detectability of CNVs was better with OCTA (40, 41). In the present study, SS-OCTA was better than ICGA for the detection of tumor-related vasculature. A total of 33 tumor vasculature lesions were not clearly detected on ICGA, and all the lesions could be overlaid with the SFVNs, and the vascular tangles corresponding to tumor vasculature appeared on SS-OCTA. Moreover, one additional tangled vascular structure, which corresponded to the connected to flat irregular CNVs on OCT B-scans, was detected by SS-OCTA. As a result, SS-OCTA may be more sensitive for the detection of tumor vasculature in CO than ICGA, and, if confirmed by others, SS-OCTA might be considered the new criterion standard for the diagnosis and monitoring of CO.

In addition, SS-OCT has shown increased thickness of the choroid in CO eyes, as well as fellow eyes. After anti-VEGF therapies in 22 eyes, the choroidal thickness (“pachychoroid”) of the affected eye showed reduction or became thinner. This finding may indicate that the origin of CO arises from the choroid rather than RPE or sclera. Furthermore, we found that the tumor size remained stable with increased tumor decalcification for a long follow-up after anti-VEGF therapy, and the tumor vasculature was relatively quiescent. Intravitreal VEGF antagonists have shown beneficial effects in CO, which is consistent with the proposed theory of osteoblast-derived VEGF playing an important role in maintaining vascular integrity and bone mass (42, 43), and anti-VEGF therapy might increase bone absorption (decalcification) of CO. Thus, the tumor vasculature of CO and the tumor control associated to the anti-VEGF agents need further study.

## Limitations

The present study has several limitations, including its cross-sectional, observational nature, a single-center research, and a relatively small sample of patients. In addition, not all the patients whom we recruited were treatment naive; others had heterogeneous treatment histories. Moreover, studies are needed to confirm our findings and determine whether tumor vasculature, anti-VEGF, and tumor decalcification correlate with tumor growth of CO or certain underlying medical factors.

## Conclusions

Using the SS-OCTA platform, we examined the SFVNs and tangled vascular blood flow properties of tumor vasculature in patients with CO. We found that tumor-related vasculature consisted of densely or loosely sea-fan vascular networks with tangled vascular structures, few at the margins of tumor or type 2 neovascularization, which is inconsistent with the proposal that tumor-related vasculature is a form of CNV rather than an SFVN structure. Further studies are needed to confirm our findings and better characterize the tumor evolution, natural history, tumor growth, tumor decalcification, and response to therapy of these different tumor vascular networks and tangled vascular structures in CO using SS-OCTA.

## Data Availability Statement

The original contributions presented in the study are included in the article/**Supplementary Material**. Further inquiries can be directed to the corresponding author.

## Ethics Statement

The studies involving human participants were reviewed and approved by Medical Ethics Committee of the Beijing Tongren Hospital. Written informed consent to participate in this study was provided by the participants’ legal guardian/next of kin. Written informed consent was obtained from the individual(s), and minor(s)’ legal guardian/next of kin, for the publication of any potentially identifiable images or data included in this article.

## Author Contributions

WW: Examination of patient, interpretation of results, and writing the manuscript. NZ: Interpretation of results and writing/reviewing the manuscript. XX: Interpretation of results and writing/reviewing the manuscript. YL: Collected the data. XP: Provided some suggestions to the manuscript. All authors contributed to the article and approved the submitted version.

## Funding

The National Natural Science Foundation of China (No. 81272981) and the Beijing Natural Science Foundation (No. 7151003) provided financial support.

## Conflict of Interest

Author XP is employed by SVision Imaging, Ltd.

The remaining authors declare that the research was conducted in the absence of any commercial or financial relationships that could be construed as a potential conflict of interest.

## Publisher’s Note

All claims expressed in this article are solely those of the authors and do not necessarily represent those of their affiliated organizations, or those of the publisher, the editors and the reviewers. Any product that may be evaluated in this article, or claim that may be made by its manufacturer, is not guaranteed or endorsed by the publisher.

## Supplementary Material

Supplementary Figure 1 (Patient 4) **(A–F)** After anti-VEGF therapy, the SS-OCTA *en face* image showed no reduction in the size of the SFVN and vascular tangles. **(G)** The terminal vascular tangles were observed to recur 2 months after anti-VEGF therapy, and the tangled vascular structure seemed more distinct in some tumor-related vasculature (eg, the tumor-related vasculature numbered A and B). **(H)** Four months after anti-VEGF therapy, denser vascular tangles were observed at the end of SFVN. They were associated with some newly formed lesions and the growth of tumor (the tumor-growth numbered A and B).

Supplementary Figure 2 (Patient 2) **(A)** Early-phase FA showed evidence of focal hyperfluorescence, and late-phase FA showed leakage with a pooling configuration that suggested the diagnosis of active retinal serous detachment. ICGA revealed focal hyperfluorescence with SFVNs in the early frames. **(B)** The tumor-related vasculature consisted of sea-fan vascular networks combined with tangled vessels, and anti-VEGF treatment was associated with regression of SRF secondary from tumor vascular structures. Patients with CO received anti-VEGF treatment, with the absorption of subretinal fluid, the choroidal thickness (“pachychoroid”) of the affected eye showed reduction or became thinner.

Supplementary Figure 3 (Patient 14) (**A**1, 2). FA showed evidence of early focal hyperfluorescence with late leakage, ICGA revealed patchy hyperfluorescence in the early frames. (**B**1, 2). On the SS-OCT, B-scan revealed a 1507.8-μm-thick on OS and 1275.7-μm-thick on OD diffused tumor, respectively. (**C**1, 2). The Montage image of superficial retinal vascular layer showed the retinal vasculature were normal.

Supplementary Figure 4 (Patient 3) **(A)** Fundus examination revealed diffused reddish-orange lesion in the superior macula. **(B)** FA showed evidence of early focal hyperfluorescence with late leakage consistent with type 2 neovascularization. ICGA revealed focal hyperfluorescence in the early frame. (**C**1, 2). The SFVN and type 2 neovascularization coexisted in the same eye, and tumor-related vasculature may have been derived from one or both of these neovascular lesions, as outlined in our schematic drawing.
