# Supplementary figures and images for: Appearance of Tumor Vessels in Patients With Choroidal Osteoma Using Swept-Source Optical Coherence Tomographic Angiography
Source: Front Oncol. 2021 Nov 1;11:762394. doi: 10.3389/fonc.2021.762394 (PMC8591199; doi:10.3389/fonc.2021.762394)

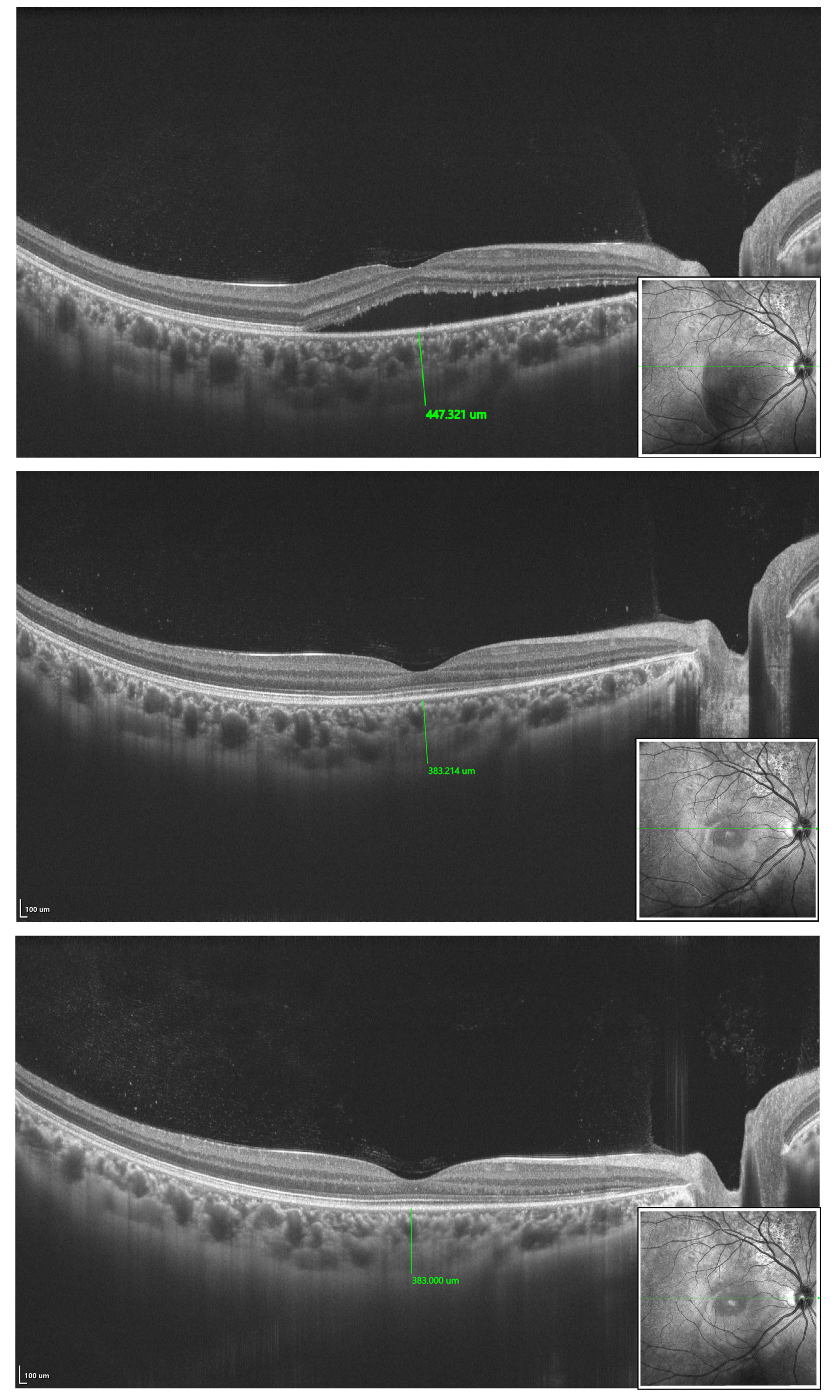

Supplement: Supplementary Figure 1 — (Patient 4) (A–F) After anti-VEGF therapy, the SS-OCTA en face image showed no reduction in the size of the SFVN and vascular tangles. (G) The terminal vascular tangles were observed to recur 2 months after anti-VEGF therapy, and the tangled vascular structure seemed more distinct in some tumor-related vasculature (eg, the tumor-related vasculature numbered A and B). (H) Four months after anti-VEGF therapy, denser vascular tangles were observed at the end of SFVN. They were associated with some newly formed lesions and the growth of tumor (the tumor-growth numbered A and B). [file DataSheet_1.zip › eFigures/eFigures-supplement of Figure 2-patient 2/eFig 2 b. OD-BSCAN-12MM-σ»╣μ»ö.tif]

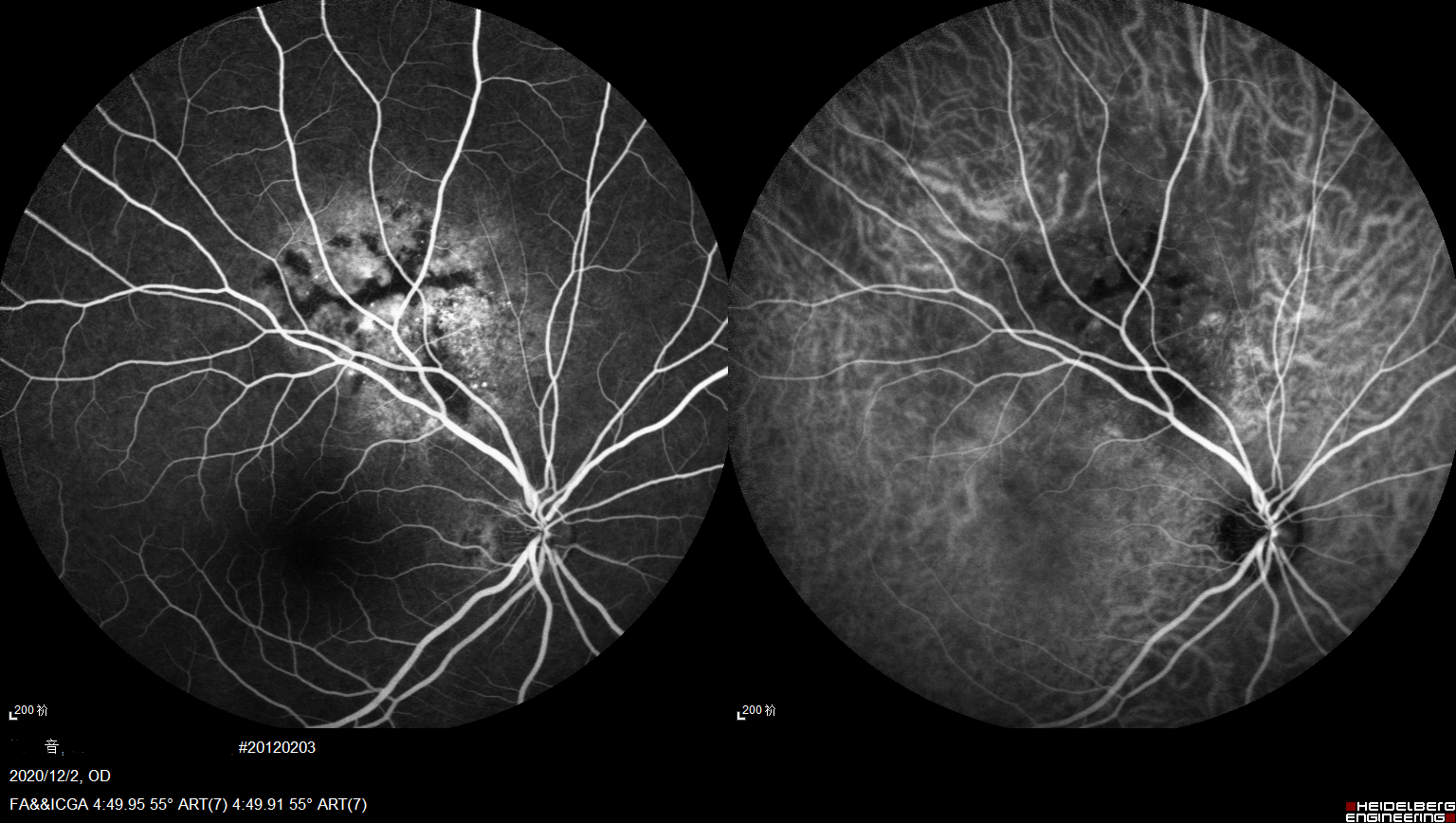

Supplement: Supplementary Figure 1 — (Patient 4) (A–F) After anti-VEGF therapy, the SS-OCTA en face image showed no reduction in the size of the SFVN and vascular tangles. (G) The terminal vascular tangles were observed to recur 2 months after anti-VEGF therapy, and the tangled vascular structure seemed more distinct in some tumor-related vasculature (eg, the tumor-related vasculature numbered A and B). (H) Four months after anti-VEGF therapy, denser vascular tangles were observed at the end of SFVN. They were associated with some newly formed lesions and the growth of tumor (the tumor-growth numbered A and B). [file DataSheet_1.zip › eFigures/eFigures-supplement of Figure 2-patient 2/eFig 2 a. τÄïΣ╜│Θƒ│w_023.tif]

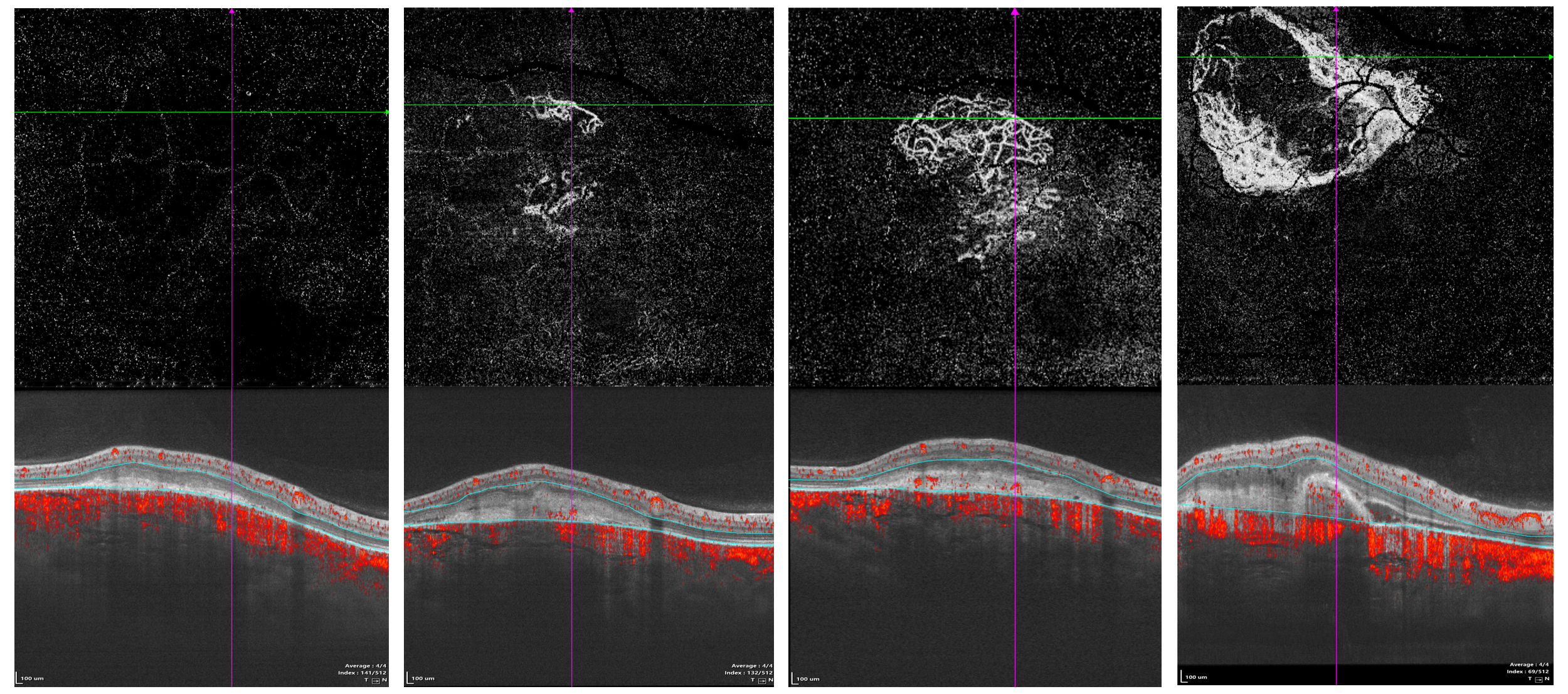

Supplement: Supplementary Figure 1 — (Patient 4) (A–F) After anti-VEGF therapy, the SS-OCTA en face image showed no reduction in the size of the SFVN and vascular tangles. (G) The terminal vascular tangles were observed to recur 2 months after anti-VEGF therapy, and the tangled vascular structure seemed more distinct in some tumor-related vasculature (eg, the tumor-related vasculature numbered A and B). (H) Four months after anti-VEGF therapy, denser vascular tangles were observed at the end of SFVN. They were associated with some newly formed lesions and the growth of tumor (the tumor-growth numbered A and B). [file DataSheet_1.zip › eFigures/efigures-supplement of Figure 4-patient 3/eFig 4c2. ΘÜÅΦ«┐σ»╣μ»ö.tif]

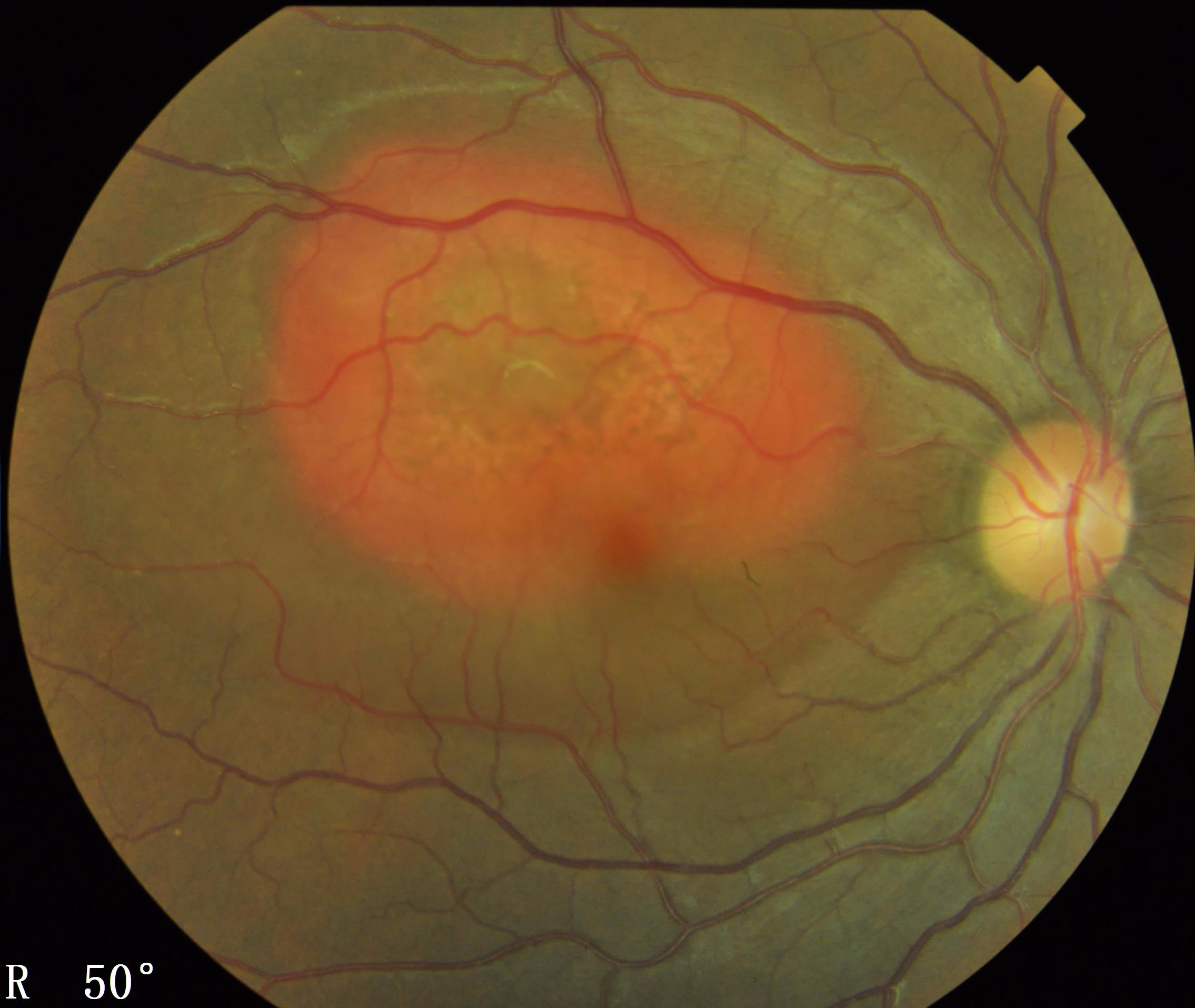

Supplement: Supplementary Figure 1 — (Patient 4) (A–F) After anti-VEGF therapy, the SS-OCTA en face image showed no reduction in the size of the SFVN and vascular tangles. (G) The terminal vascular tangles were observed to recur 2 months after anti-VEGF therapy, and the tangled vascular structure seemed more distinct in some tumor-related vasculature (eg, the tumor-related vasculature numbered A and B). (H) Four months after anti-VEGF therapy, denser vascular tangles were observed at the end of SFVN. They were associated with some newly formed lesions and the growth of tumor (the tumor-growth numbered A and B). [file DataSheet_1.zip › eFigures/efigures-supplement of Figure 4-patient 3/eFig 4a. τë¢.tif]

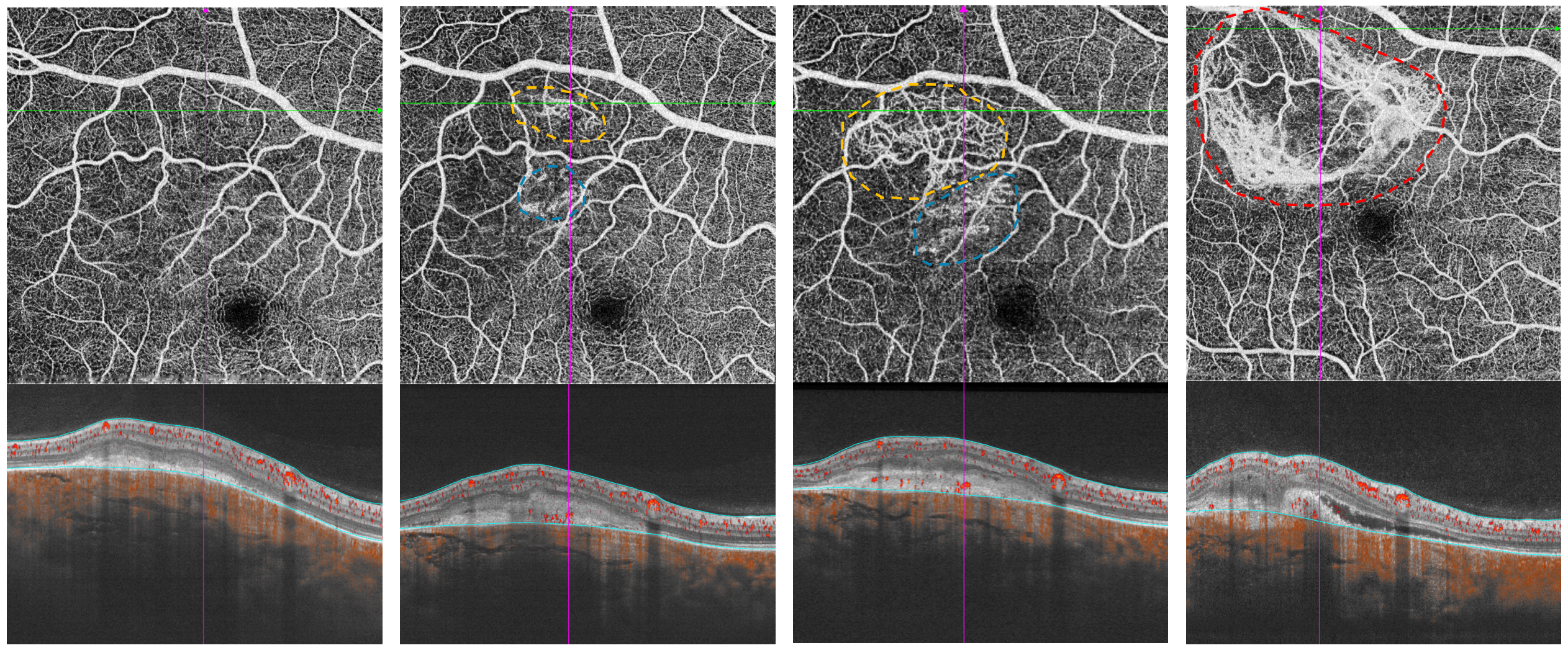

Supplement: Supplementary Figure 1 — (Patient 4) (A–F) After anti-VEGF therapy, the SS-OCTA en face image showed no reduction in the size of the SFVN and vascular tangles. (G) The terminal vascular tangles were observed to recur 2 months after anti-VEGF therapy, and the tangled vascular structure seemed more distinct in some tumor-related vasculature (eg, the tumor-related vasculature numbered A and B). (H) Four months after anti-VEGF therapy, denser vascular tangles were observed at the end of SFVN. They were associated with some newly formed lesions and the growth of tumor (the tumor-growth numbered A and B). [file DataSheet_1.zip › eFigures/efigures-supplement of Figure 4-patient 3/eFig 4c1. ΘÜÅΦ«┐σ»╣μ»ö 2.tif]

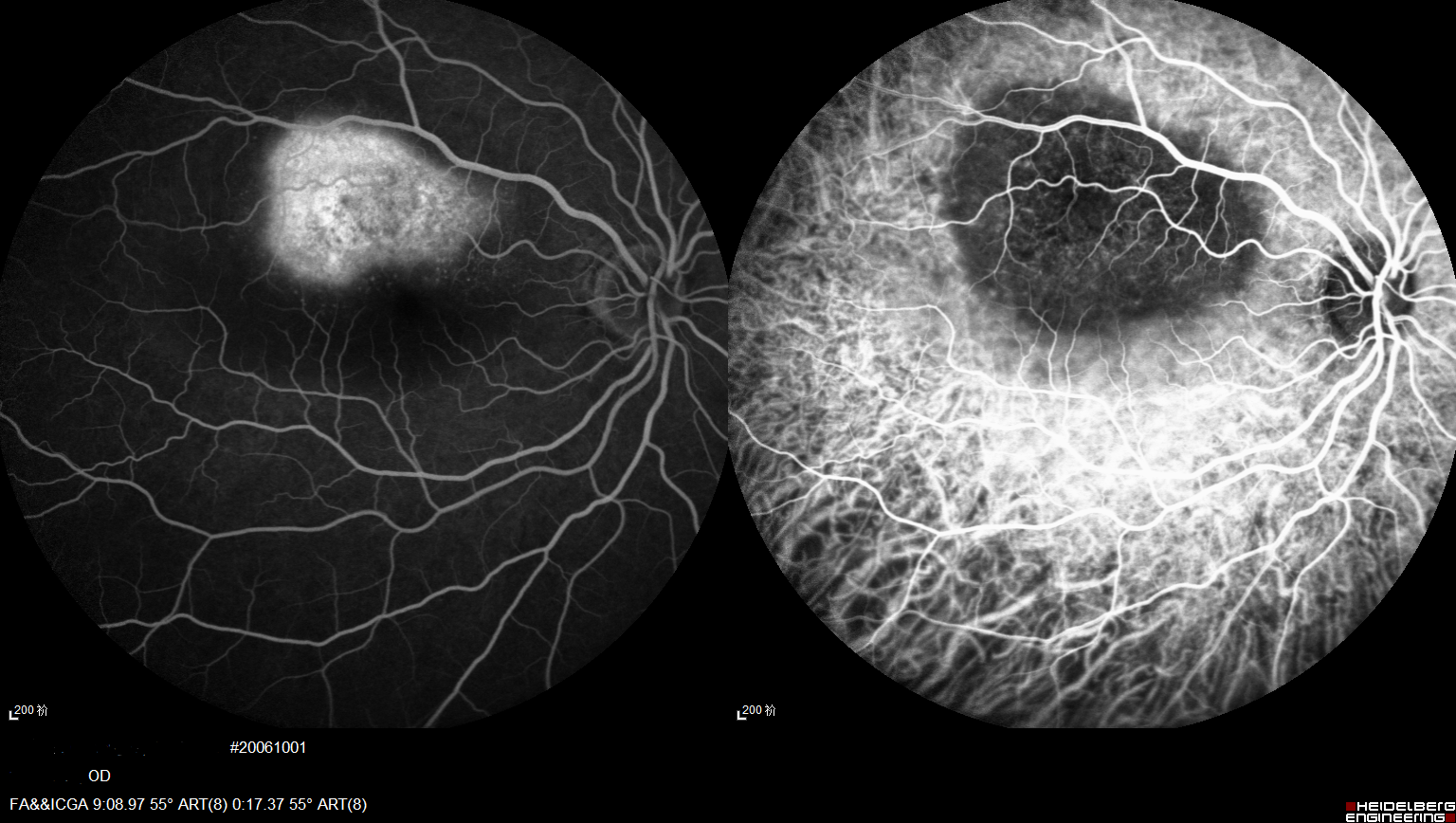

Supplement: Supplementary Figure 1 — (Patient 4) (A–F) After anti-VEGF therapy, the SS-OCTA en face image showed no reduction in the size of the SFVN and vascular tangles. (G) The terminal vascular tangles were observed to recur 2 months after anti-VEGF therapy, and the tangled vascular structure seemed more distinct in some tumor-related vasculature (eg, the tumor-related vasculature numbered A and B). (H) Four months after anti-VEGF therapy, denser vascular tangles were observed at the end of SFVN. They were associated with some newly formed lesions and the growth of tumor (the tumor-growth numbered A and B). [file DataSheet_1.zip › eFigures/efigures-supplement of Figure 4-patient 3/eFig 4b. τë¢_050.tif]

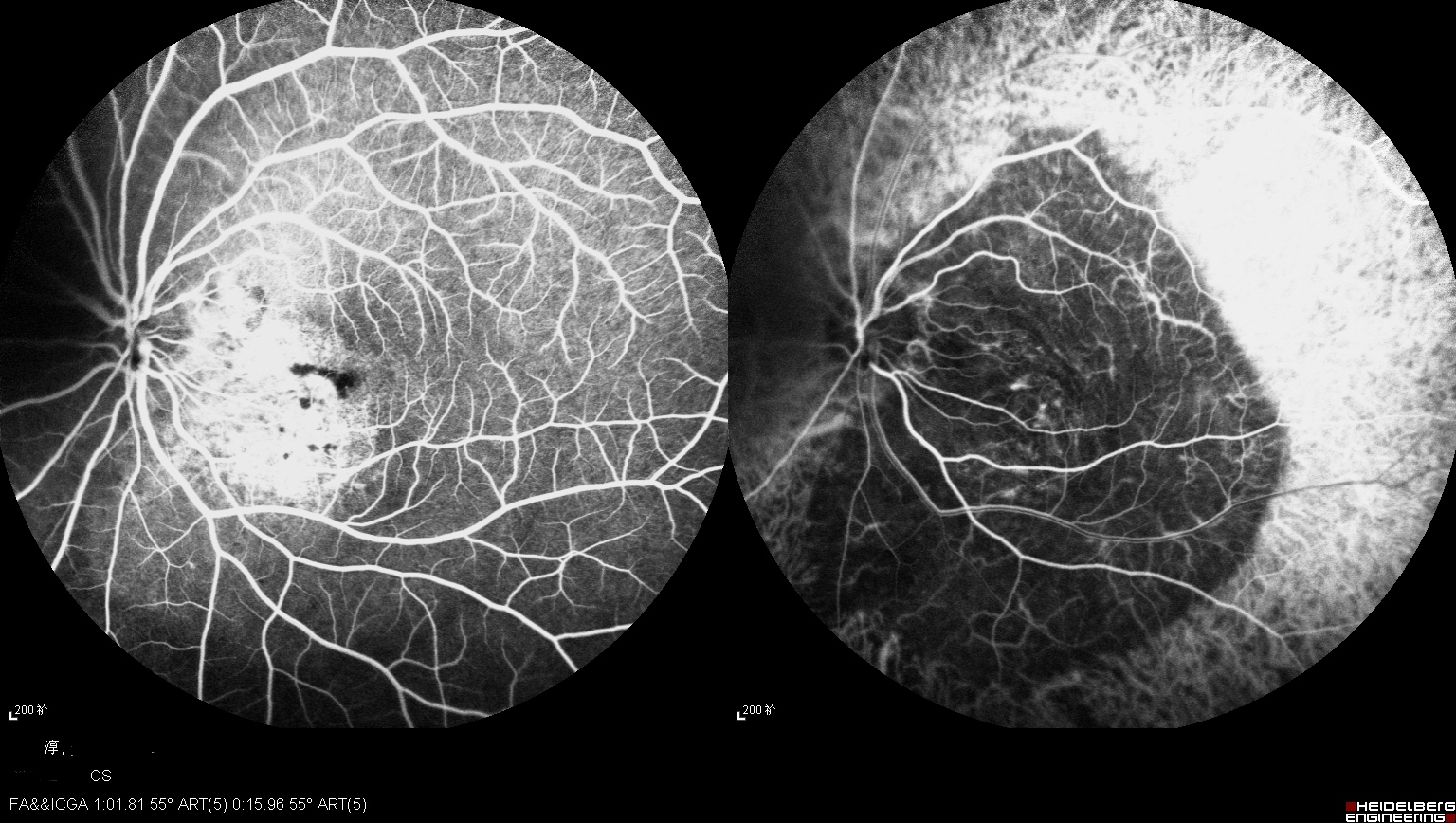

Supplement: Supplementary Figure 1 — (Patient 4) (A–F) After anti-VEGF therapy, the SS-OCTA en face image showed no reduction in the size of the SFVN and vascular tangles. (G) The terminal vascular tangles were observed to recur 2 months after anti-VEGF therapy, and the tangled vascular structure seemed more distinct in some tumor-related vasculature (eg, the tumor-related vasculature numbered A and B). (H) Four months after anti-VEGF therapy, denser vascular tangles were observed at the end of SFVN. They were associated with some newly formed lesions and the growth of tumor (the tumor-growth numbered A and B). [file DataSheet_1.zip › eFigures/efigures-supplement of Fiugre 3-patient 14/eFig 3 a2. Σ╕Ñ_033.tiff]

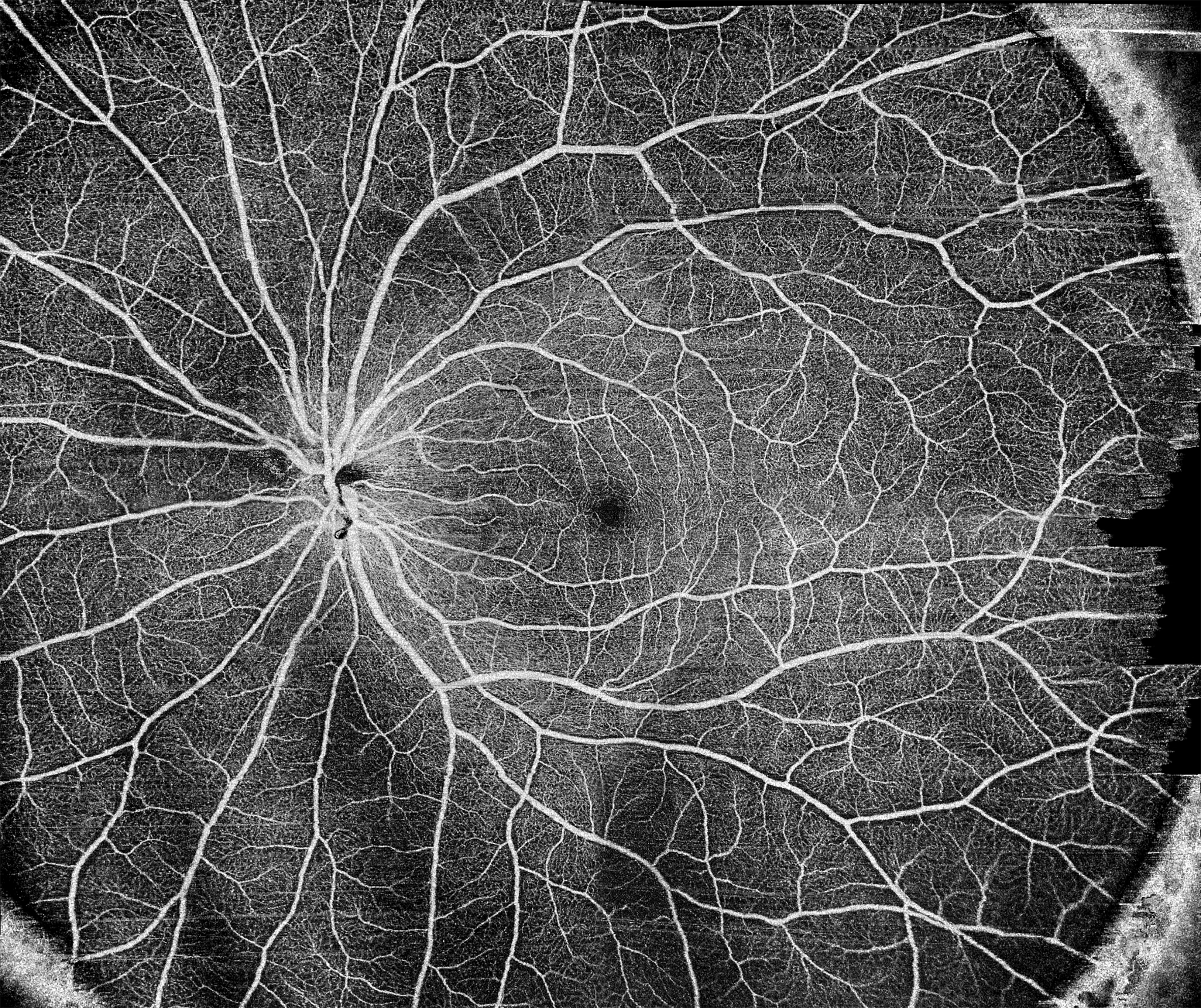

Supplement: Supplementary Figure 1 — (Patient 4) (A–F) After anti-VEGF therapy, the SS-OCTA en face image showed no reduction in the size of the SFVN and vascular tangles. (G) The terminal vascular tangles were observed to recur 2 months after anti-VEGF therapy, and the tangled vascular structure seemed more distinct in some tumor-related vasculature (eg, the tumor-related vasculature numbered A and B). (H) Four months after anti-VEGF therapy, denser vascular tangles were observed at the end of SFVN. They were associated with some newly formed lesions and the growth of tumor (the tumor-growth numbered A and B). [file DataSheet_1.zip › eFigures/efigures-supplement of Fiugre 3-patient 14/eFig 3 c2. OS-ANGIO-INNERRETINA-MANTAGE.tif]

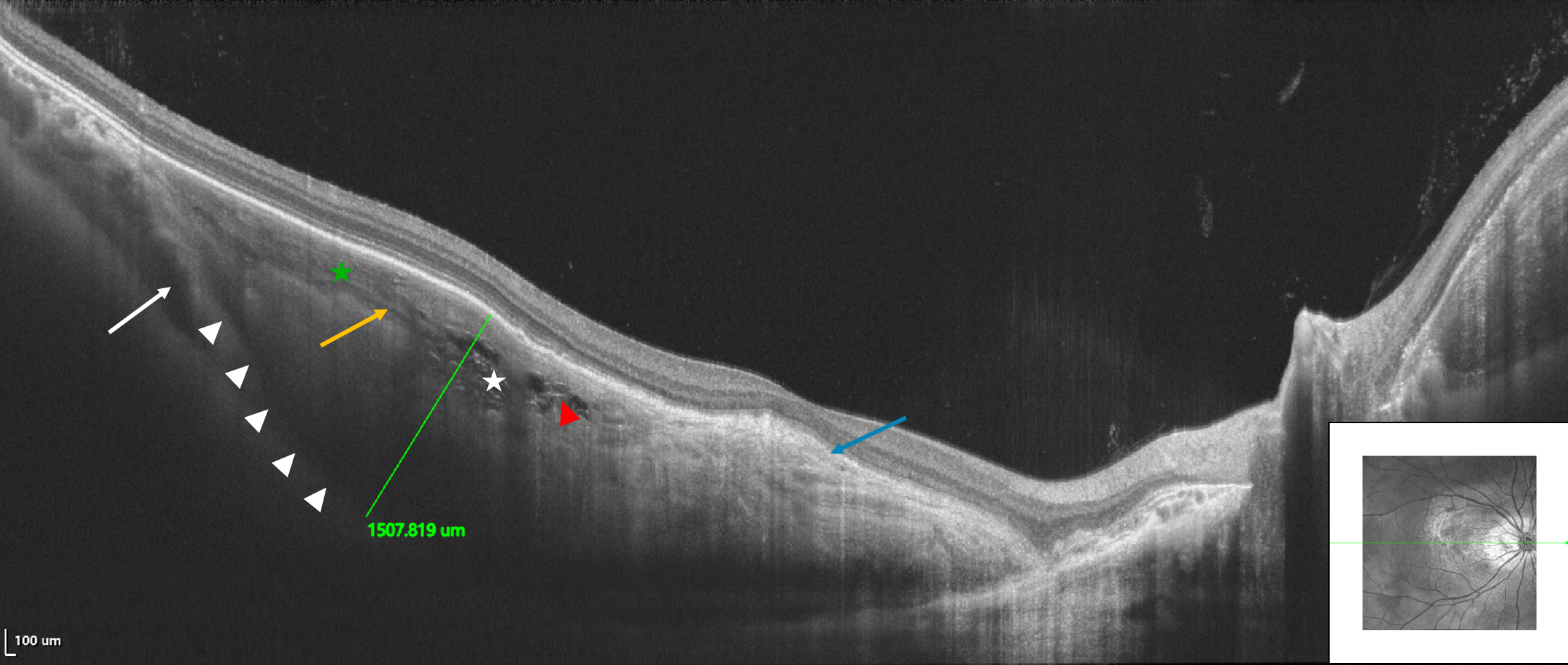

Supplement: Supplementary Figure 1 — (Patient 4) (A–F) After anti-VEGF therapy, the SS-OCTA en face image showed no reduction in the size of the SFVN and vascular tangles. (G) The terminal vascular tangles were observed to recur 2 months after anti-VEGF therapy, and the tangled vascular structure seemed more distinct in some tumor-related vasculature (eg, the tumor-related vasculature numbered A and B). (H) Four months after anti-VEGF therapy, denser vascular tangles were observed at the end of SFVN. They were associated with some newly formed lesions and the growth of tumor (the tumor-growth numbered A and B). [file DataSheet_1.zip › eFigures/efigures-supplement of Fiugre 3-patient 14/eFig 3 b1. OD-BSCAN-16MM.tif]

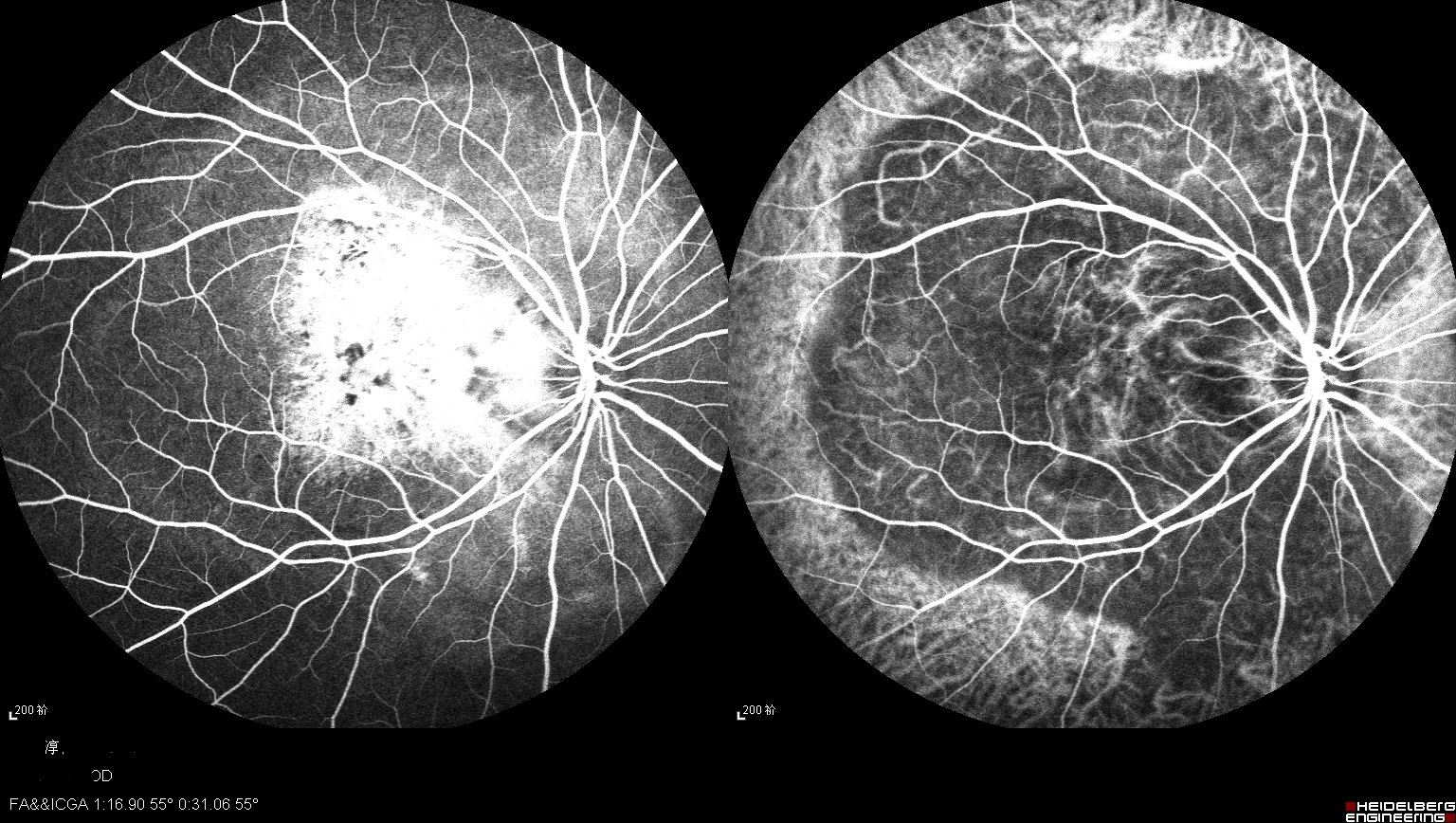

Supplement: Supplementary Figure 1 — (Patient 4) (A–F) After anti-VEGF therapy, the SS-OCTA en face image showed no reduction in the size of the SFVN and vascular tangles. (G) The terminal vascular tangles were observed to recur 2 months after anti-VEGF therapy, and the tangled vascular structure seemed more distinct in some tumor-related vasculature (eg, the tumor-related vasculature numbered A and B). (H) Four months after anti-VEGF therapy, denser vascular tangles were observed at the end of SFVN. They were associated with some newly formed lesions and the growth of tumor (the tumor-growth numbered A and B). [file DataSheet_1.zip › eFigures/efigures-supplement of Fiugre 3-patient 14/eFig 3 a1. Σ╕Ñ_006.tiff]

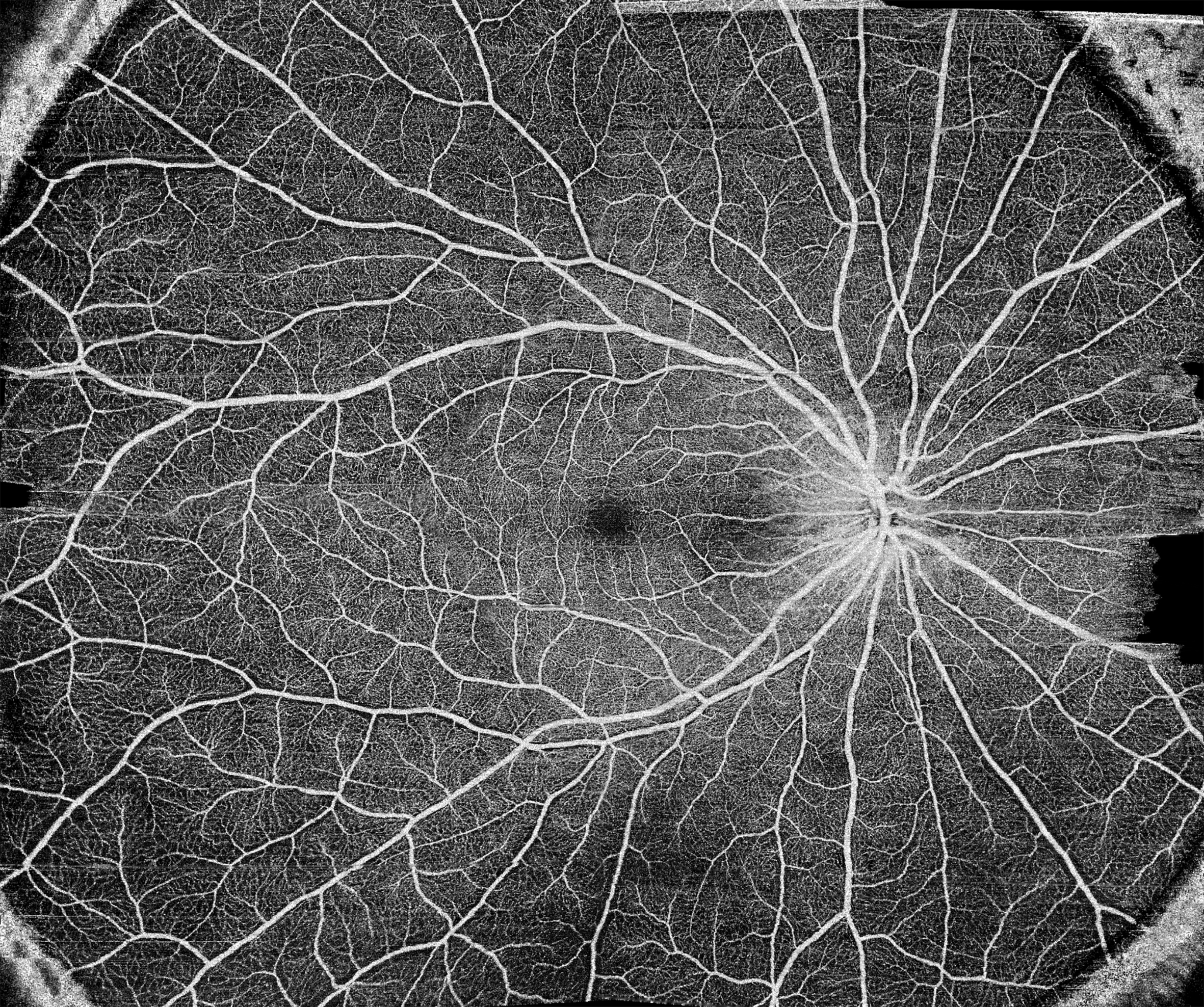

Supplement: Supplementary Figure 1 — (Patient 4) (A–F) After anti-VEGF therapy, the SS-OCTA en face image showed no reduction in the size of the SFVN and vascular tangles. (G) The terminal vascular tangles were observed to recur 2 months after anti-VEGF therapy, and the tangled vascular structure seemed more distinct in some tumor-related vasculature (eg, the tumor-related vasculature numbered A and B). (H) Four months after anti-VEGF therapy, denser vascular tangles were observed at the end of SFVN. They were associated with some newly formed lesions and the growth of tumor (the tumor-growth numbered A and B). [file DataSheet_1.zip › eFigures/efigures-supplement of Fiugre 3-patient 14/eFig 3 c1. OD-ANGIO-INNERRETINA-MANTAGE.tif]

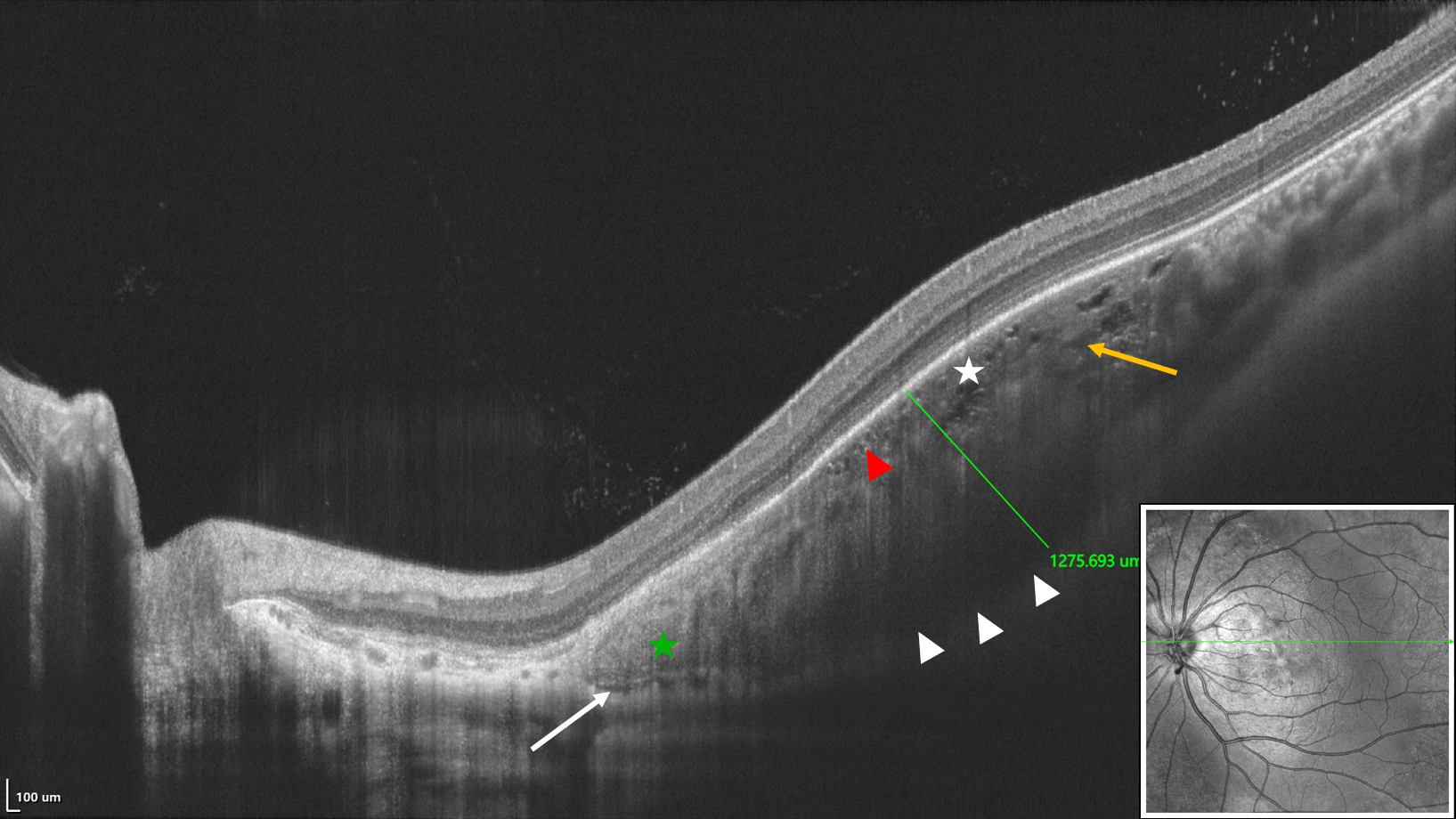

Supplement: Supplementary Figure 1 — (Patient 4) (A–F) After anti-VEGF therapy, the SS-OCTA en face image showed no reduction in the size of the SFVN and vascular tangles. (G) The terminal vascular tangles were observed to recur 2 months after anti-VEGF therapy, and the tangled vascular structure seemed more distinct in some tumor-related vasculature (eg, the tumor-related vasculature numbered A and B). (H) Four months after anti-VEGF therapy, denser vascular tangles were observed at the end of SFVN. They were associated with some newly formed lesions and the growth of tumor (the tumor-growth numbered A and B). [file DataSheet_1.zip › eFigures/efigures-supplement of Fiugre 3-patient 14/eFig 3 b2. OS-BSCAN-12MM.tif]

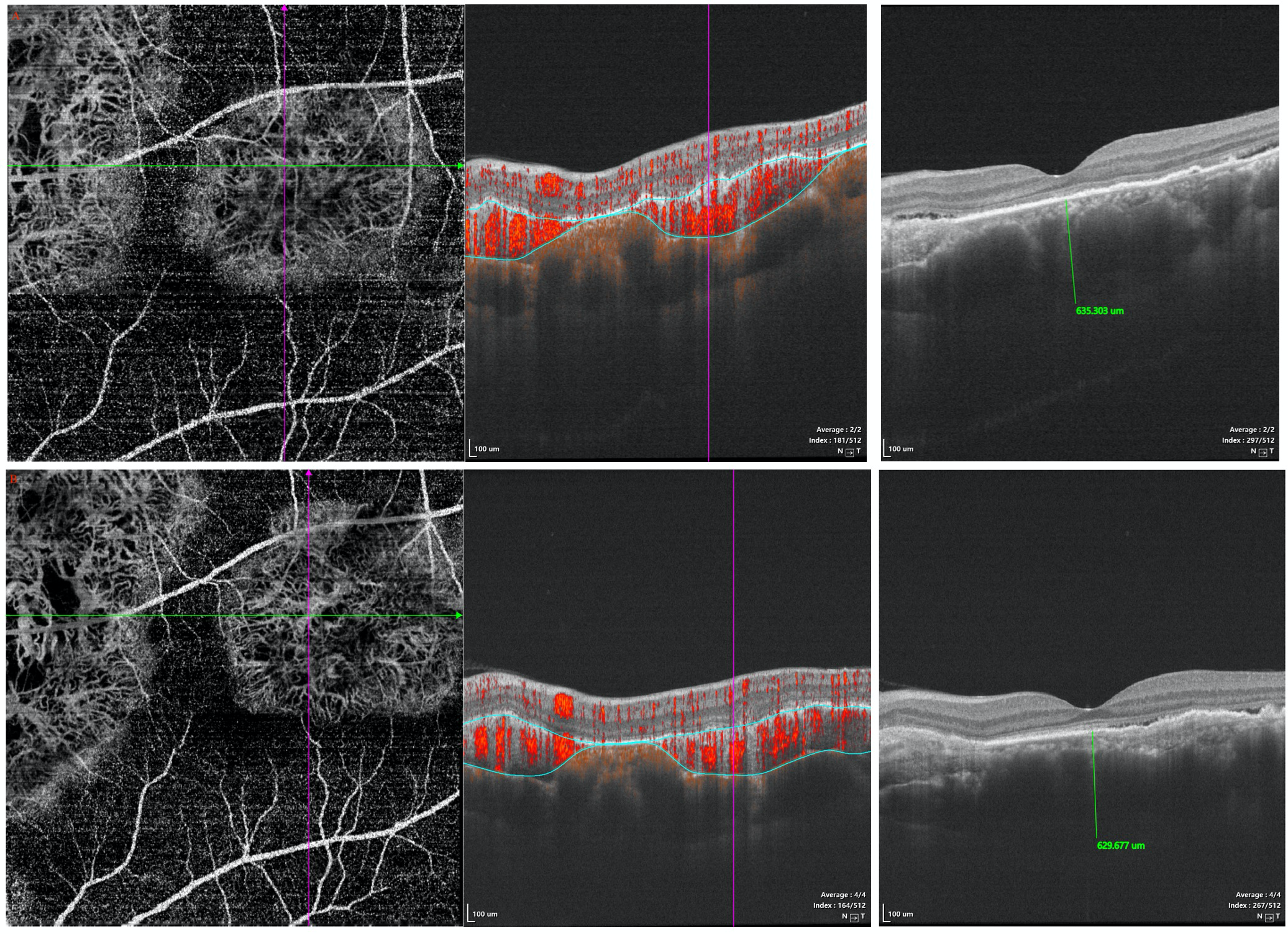

Supplement: Supplementary Figure 1 — (Patient 4) (A–F) After anti-VEGF therapy, the SS-OCTA en face image showed no reduction in the size of the SFVN and vascular tangles. (G) The terminal vascular tangles were observed to recur 2 months after anti-VEGF therapy, and the tangled vascular structure seemed more distinct in some tumor-related vasculature (eg, the tumor-related vasculature numbered A and B). (H) Four months after anti-VEGF therapy, denser vascular tangles were observed at the end of SFVN. They were associated with some newly formed lesions and the growth of tumor (the tumor-growth numbered A and B). [file DataSheet_1.zip › eFigures/eFigures-supplement of Figure 1-patient 4/eFig 1 g.tiff]

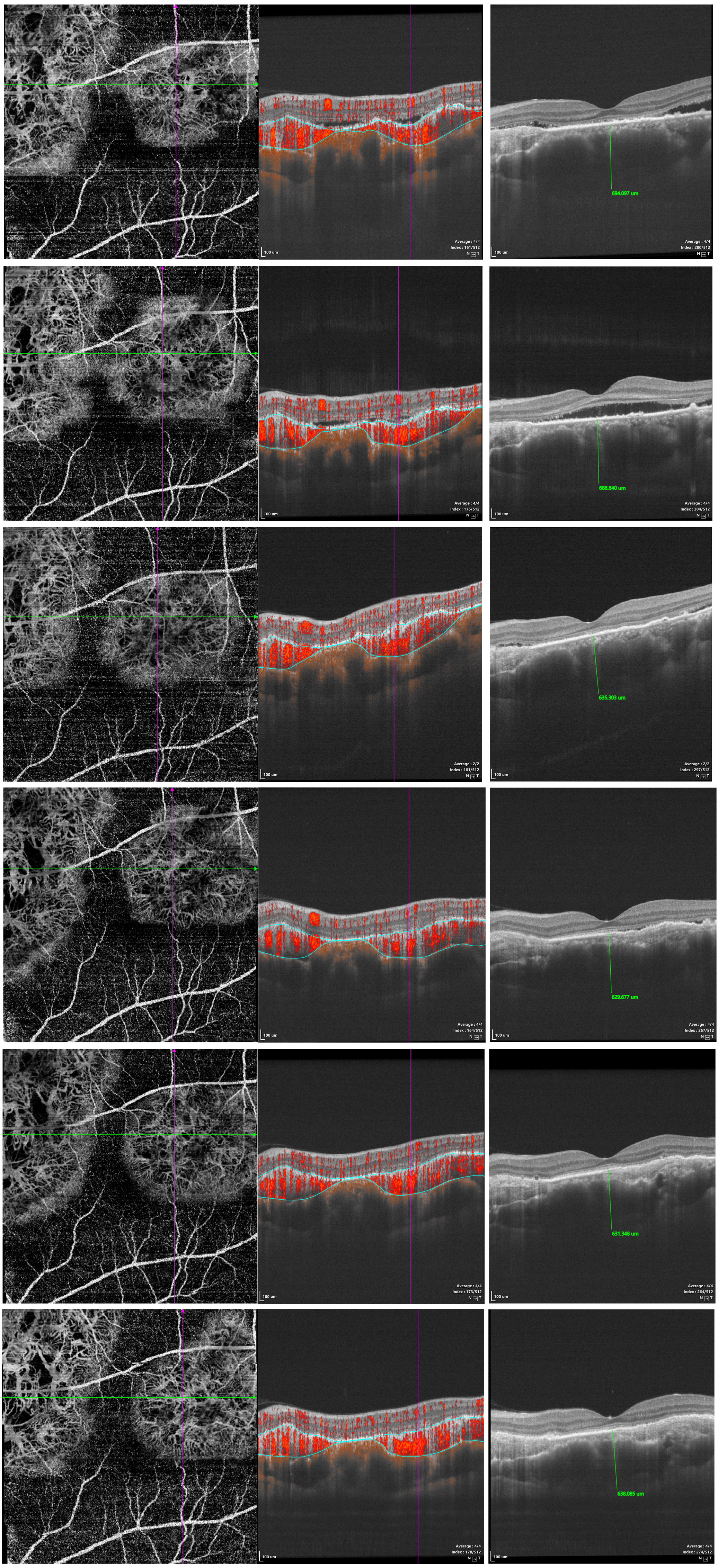

Supplement: Supplementary Figure 1 — (Patient 4) (A–F) After anti-VEGF therapy, the SS-OCTA en face image showed no reduction in the size of the SFVN and vascular tangles. (G) The terminal vascular tangles were observed to recur 2 months after anti-VEGF therapy, and the tangled vascular structure seemed more distinct in some tumor-related vasculature (eg, the tumor-related vasculature numbered A and B). (H) Four months after anti-VEGF therapy, denser vascular tangles were observed at the end of SFVN. They were associated with some newly formed lesions and the growth of tumor (the tumor-growth numbered A and B). [file DataSheet_1.zip › eFigures/eFigures-supplement of Figure 1-patient 4/eFig 1 a-f.tif]

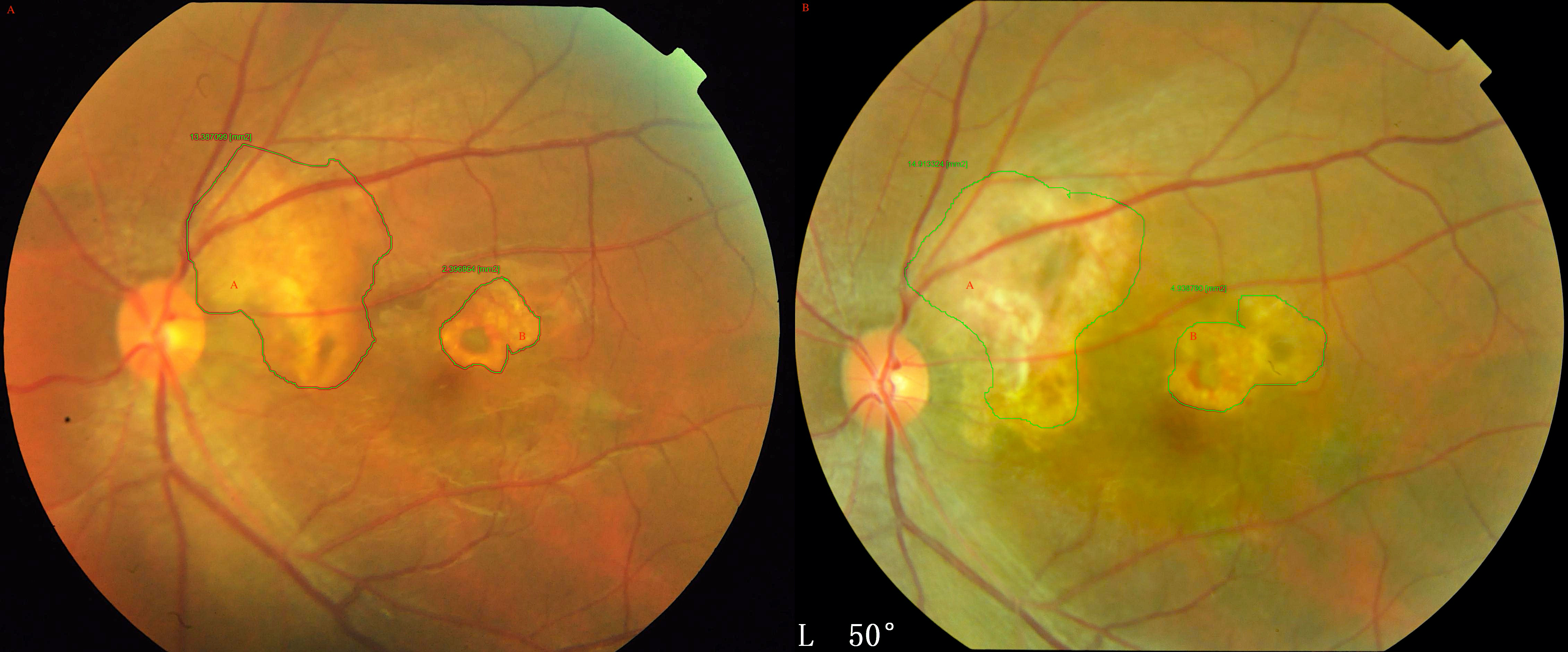

Supplement: Supplementary Figure 1 — (Patient 4) (A–F) After anti-VEGF therapy, the SS-OCTA en face image showed no reduction in the size of the SFVN and vascular tangles. (G) The terminal vascular tangles were observed to recur 2 months after anti-VEGF therapy, and the tangled vascular structure seemed more distinct in some tumor-related vasculature (eg, the tumor-related vasculature numbered A and B). (H) Four months after anti-VEGF therapy, denser vascular tangles were observed at the end of SFVN. They were associated with some newly formed lesions and the growth of tumor (the tumor-growth numbered A and B). [file DataSheet_1.zip › eFigures/eFigures-supplement of Figure 1-patient 4/eFig 1 h.tiff]
